# Lysosomal-Associated Protein Multispanning Transmembrane *5* Gene (*LAPTM5*) Is Associated with Spontaneous Regression of Neuroblastomas

**DOI:** 10.1371/journal.pone.0007099

**Published:** 2009-09-29

**Authors:** Jun Inoue, Akiko Misawa, Yukichi Tanaka, Shizuko Ichinose, Yuriko Sugino, Hajime Hosoi, Tohru Sugimoto, Issei Imoto, Johji Inazawa

**Affiliations:** 1 Department of Molecular Cytogenetics, Medical Research Institute and School of Biomedical Science, Tokyo Medical and Dental University, Tokyo, Japan; 2 Instrumental Analysis Research Center, Tokyo Medical and Dental University, Tokyo, Japan; 3 Hard Tissue Genome research Center, Tokyo Medical and Dental University, Tokyo, Japan; 4 21st Century Center of Excellence Program for Molecular Destruction and Reconstitution of Tooth and Bone, Tokyo Medical and Dental University, Tokyo, Japan; 5 Global Center of Excellence (GCOE) Program, International Research Center for Molecular Science in Tooth and Bone Disease, Saitama, Japan; 6 Core Research for Evolutionary Science and Technology of the Japan Science and Technology Corporation, Saitama, Japan; 7 Division of Pathology, Kanagawa Children's Medical Center, Kanagawa, Japan; 8 Department of Pediatrics, Graduate School of Medical Science, Kyoto Prefectural University of Medicine, Kyoto, Japan; 9 Saiseikai Shiga Hospital, Shiga, Japan; Technical University Munich, Germany

## Abstract

**Background:**

Neuroblastoma (NB) is the most frequently occurring solid tumor in children, and shows heterogeneous clinical behavior. Favorable tumors, which are usually detected by mass screening based on increased levels of catecholamines in urine, regress spontaneously via programmed cell death (PCD) or mature through differentiation into benign ganglioneuroma (GN). In contrast, advanced-type NB tumors often grow aggressively, despite intensive chemotherapy. Understanding the molecular mechanisms of PCD during spontaneous regression in favorable NB tumors, as well as identifying genes with a pro-death role, is a matter of urgency for developing novel approaches to the treatment of advanced-type NB tumors.

**Principal Findings:**

We found that the expression of *lysosomal associated protein multispanning transmembrane 5* (*LAPTM5*) was usually down-regulated due to DNA methylation in an NB cell-specific manner, but up-regulated in degenerating NB cells within locally regressing areas of favorable tumors detected by mass-screening. Experiments *in vitro* showed that not only a restoration of its expression but also the accumulation of LAPTM5 protein, was required to induce non-apoptotic cell death with autophagic vacuoles and lysosomal destabilization with lysosomal-membrane permeabilization (LMP) in a caspase-independent manner. While autophagy is a membrane-trafficking pathway to degrade the proteins in lysosomes, the LAPTM5-mediated lysosomal destabilization with LMP leads to an interruption of autophagic flux, resulting in the accumulation of immature autophagic vacuoles, p62/SQSTM1, and ubiqitinated proteins as substrates of autophagic degradation. In addition, ubiquitin-positive inclusion bodies appeared in degenerating NB cells.

**Conclusions:**

We propose a novel molecular mechanism for PCD with the accumulation of autophagic vacuoles due to LAPTM5-mediated lysosomal destabilization. LAPTM5-induced cell death is lysosomal cell death with impaired autophagy, not cell death by autophagy, so-called autophagic cell death. Thus LAPTM5-mediated PCD is closely associated with the spontaneous regression of NBs and opens new avenues for exploring innovative clinical interventions for this tumor.

## Introduction

Neuroblastoma (NB) is a malignant tumor consisting of undifferentiated neuroectodermal cells from the neural crest and the most common solid tumor in children. Most primary tumors occur within the abdomen, with the adrenal medulla affected in about 50% of NB patients [1]–[3]. The clinical behavior of NB is heterogeneous: favorable tumors, which are usually detected by mass screening based on increased levels of catecholamines in urine, regress spontaneously via programmed cell death (PCD) or mature through differentiation into benign ganglioneuromas (GN) in patients under 1 year of age, with minimal or no therapeutic intervention; while advanced-type NBs, usually clinically detected, often grow rapidly to become fatal in older children, despite intensive chemotherapy [1]–[3].

The spontaneous regression and maturation of favorable NBs coincide with the neuronal differentiation and massive cellular suicide during the normal development of the nervous system, indicating that NB cells in favorable tumors may retain the genetic program of their normal counterparts [2]. Therefore, it has been considered that NB cells within regressing tumors may undergo apoptosis (a form of PCD), involving the neurotrophin signaling pathway through TrkA (receptor) and nerve growth factor (NGF) [3]–[9]. However some groups failed to find evidence of a correlation between apoptosis and factors associated with spontaneous regression [10]–[12]. On the other hand, a previous electron microscopic analysis revealed that degenerative changes including the accumulation of autophagic vacuoles are a conspicuous feature of NBs [13], and it has also been proposed that the spontaneous regression of NBs may occur via a H-Ras-associated non-apoptotic mechanism with the appearance of autophagic vacuoles in a caspase-independent manner [12]. Thus, evidence suggests that both apoptosis and non-apoptotic cell death play a role in the spontaneous regression of NBs [5].

The “wait and see” strategy has revealed that one third of mass-screened NB tumors undergo spontaneous regression, and a complete regression usually takes place over 4–20 months [4], [14]–[17]. This slow process suggests that the degenerating NB cells in the late phase of PCD are a small proportion in favorable NB tumors found by mass-screening and then exenterated surgically, and therefore some genes having a pro-death role in PCD may yet to be expressed in mass-screened NB tumor samples. To develop a breakthrough in therapy against advanced-type NBs, which can not regress spontaneously, it is important to identify genes having a critical role in spontaneous regression/maturation and to understand those molecular mechanisms.

Here, we found *lysosomal associated protein multispanning transmembrane 5* (*LAPTM5*) to be closely associated with the spontaneous regression of mass-screened NB tumors. Whereas the expression of this gene was usually down-regulated through DNA methylation in favorable and unfavorable NB tumor samples, it was up-regulated in degenerating NB cells within locally regressing areas of mass-screened tumors. Moreover, overexpression of this gene induced caspase-independent lysosomal cell death due to lysosomal destabilization with lysosomal-membrane permeabilization (LMP), with the accumulation of immature autophagic vacuoles and ubiquitinated proteins, leading us to propose novel characteristics for PCD during the spontaneous regression of NBs.

## Results

### Down-regulation of *LAPTM5* expression through DNA methylation in NB cells

Previously, we developed a DNA methylation-screening system, bacterial artificial chromosome (BAC) array-based methylated CpG island amplification (MCA) [18] (BAMCA) [19], and identified tumor-related genes down-regulated through DNA methylation in human cancers, including neuroblastoma [20]–[22]. Using this system with an in-house “1p35–p36 contig BAC-array” [19], we identified one BAC at 1p35 as potentially methylated in NB cell lines, by comparing with two NB cell lines (GOTO and IMR32) and a reference (normal peripheral blood mononuclear cells, PBMNCs) (**Supplementary Figure S1, Supplementary Table S1**, and **Supplementary Methods S1**). Screening for two candidate genes (*MATN1* and *LAPTM5*) on this BAC revealed that the expression of *LAPTM5* was down-regulated in NB cell lines and the CG sites around the transcriptional start site (TSS) of this gene were highly and widely methylated in both NB cell lines and primary NB tumors (**Supplementary Figure S1, Supplementary Table S1**, and **Supplementary Methods S1**), prompting us to focus on *LAPTM5* as a candidate for an NB-related gene down-regulated through DNA methylation.

**Figure 1:**
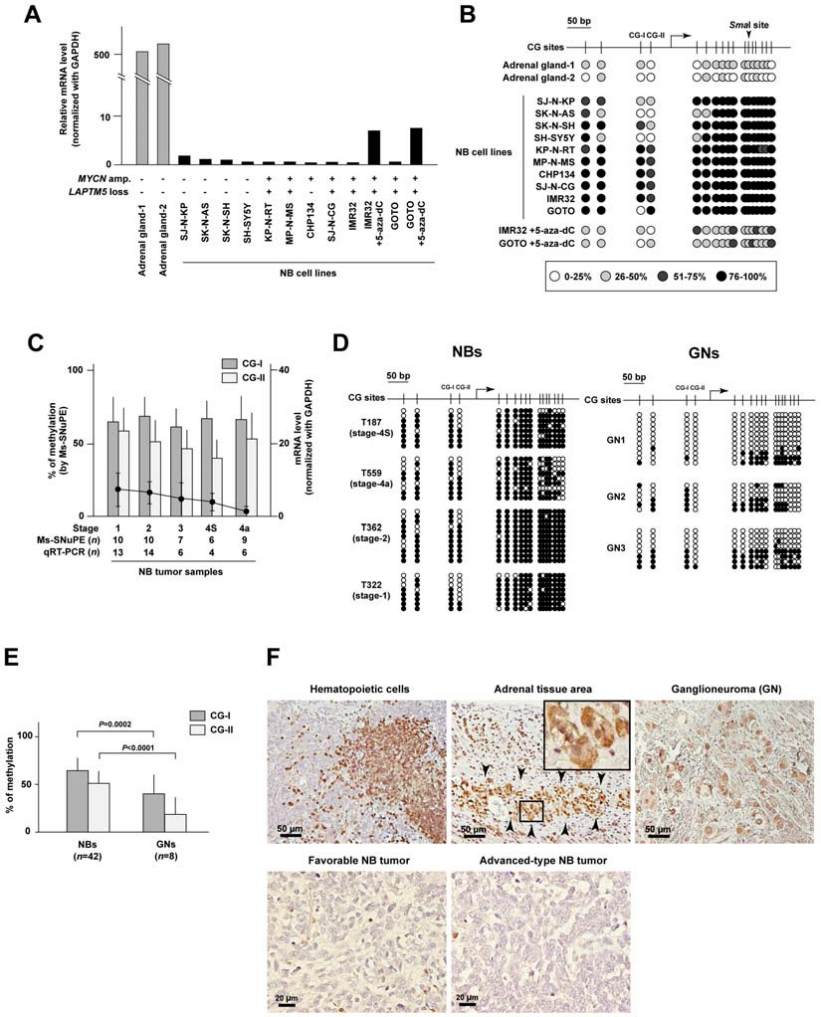
Identification of methylated DNA and down-regulation of *LAPTM5* expression in NB cell lines and primary NB tumors. (A) *LAPTM5* mRNA levels in NB cell lines with or without *MYCN* amplification (amp.) and/or allelic loss of the *LAPTM5* gene were determined by quantitative RT-PCR (qRT-PCR) and are indicated as bars. Levels of *LAPTM5* mRNA were normalized with levels of *GAPDH* mRNA. GOTO and IMR32 cells were treated with 5-aza-2′-deoxycytidine (5-aza-dC, 10 µM for 5 days). (B) Frequencies of methylation at 17 CG sites within region-I around the transcriptional-start site (TSS) of the *LAPTM5* gene were determined by bisulfite sequencing, as indicated in Supplementary Figure S1. An arrow indicates the TSS. (C) Methylation frequency and mRNA level of *LAPTM5* in primary NB tumors at different stages. Levels of *LAPTM5* mRNA measured by qRT-PCR are indicated as dots; whereas methylation frequencies at two CG sites (CG-I or –II) measured by Ms-SNuPE are indicated as dark gray and light bars, respectively. Levels of *LAPTM5* mRNA were normalized with levels of *GAPDH* mRNA. *n* indicates the number of cases. Vertical lines, SD. (D) Representative results of bisulfite sequencing at CG sites around the TSS of *LAPTM5* in primary NB tumors and ganglioneuromas (GNs). Methylation status is indicated as white (unmethylated) or black (methylated) circles. An arrow indicates the TSS. (E) Methylation frequency of *LAPTM5* in primary NB tumors and GNs. The methylation frequencies at two CG site in 8 GNs were measured by Ms-SNuPE. The average frequency of methylation in primary NB tumors or GNs is indicated. *n* indicates the number of cases. Vertical lines, SD. (F) Representative images of immunostaining for LAPTM5 in tissue sections. *Upper*; representative images of hematopoietic cells, adrenal tissue within tumor sections, and GN. The enlarged image shows the adrenal medulla cells within adrenal tissue indicated by arrowheads. *Lower*; representative images of NB cells within a favorable tumor (stage-1) detected by mass-screening and an advanced-type tumor (stage-4a) with *MYCN* amplification.

First, we examined the mRNA level, frequency of methylation, and copy-number status of the *LAPTM5* gene in 10 NB cell lines. The expression of *LAPTM5* was down-regulated in all the cell lines and the down-regulation was inversely correlated with methylation at the CG sites around the TSS, compared with normal adrenal glands (**Figure 1A and 1B**), regardless of *MYCN* amplification or allelic loss of *LAPTM5 (* **Supplementary Figure S2, Supplementary Table S2**, and **Supplementary Methods S1**). In addition, expression of *LAPTM5* was restored in correlation with a decrease of the methylation level after treatment with 5-aza-2′-deoxycytidine, a demethylating drug, in IMR32 and GOTO cells, although the expression level of LAPTM5 after the restoration was lower than that in normal adrenal glands (**Figure 1A, 1B**). We next determined the mRNA level by quantitative RT-PCR and the frequency of methylation at two CG sites (CG-I and –II) upstream of TSS of *LAPTM5* by the methylation-sensitive single nucleotide primer extension (Ms-SNuPE) method in primary NB tumors, including favorable (stage-1, -2, -3 and 4S) tumors detected by mass screening and unfavorable (stage-4a) tumors (**Supplementary Figure S3**). Methylation was detected in all primary NB tumors examined and there was no significant difference in mRNA level and methylation status among tumors at each stage (**Figure 1C**). In addition, bisulfite sequencing revealed that the CG sites around the TSS of *LAPTM5* were highly and widely methylated in NB tumors and frequently demethylated in benign ganglioneuromas (GNs) that had developed from NB tumors (**Figure 1D**). Notably, the frequency of methylation at the two CG sites, especially CG-II, was significantly decreased in GNs compared with NB tumors (66.4% to 43.0% for CG-I; *p* = 0.0002, 50.3% to 20.9% for CG-II; *p*<0.0001; **Figure 1E**). Furthermore, we examined the level of LAPTM5 protein in NB tumor sections by conducting an immunohistochemical analysis using a specific antibody and confirmed that the protein is highly expressed in hematopoietic cells as reported [23] (**Figure 1F**). Although LAPTM5 was also highly expressed in adjacent adrenal medulla cells within the tumor sections and weakly expressed in differentiated ganglion cells within GNs, its immunoreactivity in NB cells was much lower, regardless of the prognosis (**Figure 1F**). Taken together, these results suggested the expression of *LAPTM5* to be epigenetically down-regulated through DNA methylation in all NB cell lines and primary NB tumor samples and these conditions to be associated with the differentiation of tumors. Thus, the methylation and down-regulation of *LAPTM5* may be NB cell lineage-specific.

### Up-regulation of LAPTM5 expression in degenerating NB cells within regressing areas of mass-screened NB tumors

**Figure 2:**
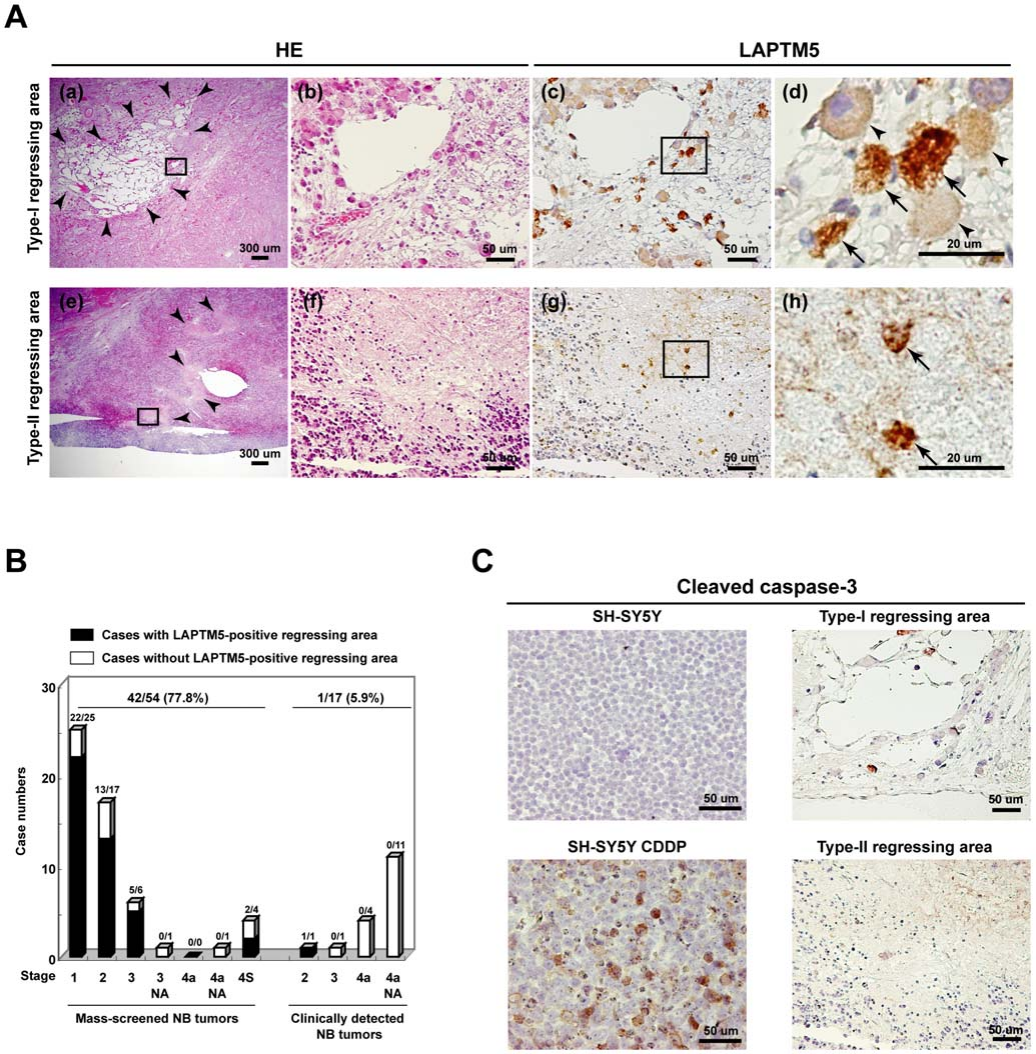
Immunostaining of LAPTM5 protein in degenerating NB cells within regressing area in mass-screened NB tumors. (A) Serial tumor sections were stained with hematoxylin-eosin (HE) (a, b, e, and f) or with anti-LAPTM5 (c, d, g, and h). a–d; representative image of a type-I regressing area, with a loss of cells including differentiating NB cells. e–h; representative image of a type-II regressing area, with a large loss of undifferentiated NB cells. The images of b, d, f, and h are the areas enlarged by the rectangle in a, c, e, and g, respectively. The area surrounded with arrowheads in a and e indicates a typical regressing area. In d and h images, arrowheads and arrows indicate differentiating NB cells and degenerating NB cells, respectively. (B) Relationship of between LAPTM5-associated regression with patients and tumor characteristics. A comparison was made of the proportion of positive cases between mass-screened NBs and clinically detected NBs. The stage of each tumor was determined using the INSS (International Neuroblastoma Staging System). NA; cases with *MYCN* amplification. (C) Representative image of immunostaining of cleaved caspase-3. SH-SY5Y cells with or without treatment with Cisplatin (CDDP; 1.5 µg/ml) for 2 days or serial tumor sections were stained with an antibody to cleaved caspase-3 antibody.

A focal regressing area is defined as a shrinking locus, accompanied by gaps or clefts within a tumor section, which occasionally appears in mass-screened NB tumors [12]. As shown in **Figure 2A**, we found two types of regressing areas on Hematoxylin-Eosin (HE) staining; type-I defined by a loss of cells within the area including a number of differentiating NB cells with an eosinophilic and large cytoplasm, and type-II defined by the loss of a large group of undifferentiating NB cells. Interestingly, our immunohistochemical analysis revealed the LAPTM5 protein to be highly expressed in the NB cells within regressing areas (the degenerating NB cells) (**Figure 2A**). We further confirmed a report [12] that H-Ras was also highly expressed in such areas (**Supplementary Figure S4**). Notably, while LAPTM5 was weakly expressed in the differentiating NB cells within type-I regressing areas, it appeared to be accumulated in the degenerating NB cells within type-I and –II regressing areas, compared with that in differentiating NB cells, adrenal medulla cells, or ganglion cells in GNs (**Figure 1F** and **Figure 2A**). These regressing areas with LAPTM5-positive degenerating NB cells were much more frequently observed in the mass-screened NB tumors (42 of 54 tumors, 77.8%), than in the clinically detected NB tumors (1 of 17 tumors, 5.9%) (**Figure 2B**). Furthermore, only one clinically detected tumor with LAPTM5-positive degenerating areas was classified as favorable (stage-2, well-differentiating). In addition, the immunostaining of cleaved caspase-3 revealed that LAPTM5-positive degenerating NB cells were essentially negative at least when the cleaved caspase-3 was present in SH-SY5Y cells induced into apoptosis by treatment with Cisplatin (CDDP) (**Figure 2C**), suggesting that caspase-independent cell death might occur in LAPTM5-positive degenerating NB cells. We further confirmed that the hematopoietic cells expressing the CD20 antigen (BCA-B20) specific for normal peripheral B lymphocytes could not be detected within the regressing areas of mass-screened NB tumors (**Supplementary Figure S4**). Those results suggest that the expression of LAPTM5 is closely associated with the spontaneous regression of mass-screened NB tumors and the accumulation of this protein rather than the restoration of its expression contributes to the PCD of NB cells in a caspase-independent manner.

### LAPTM5 overexpression induces caspase-independent cell death and the accumulation of this protein is required to induce cell death in NB cell lines

**Figure 3:**
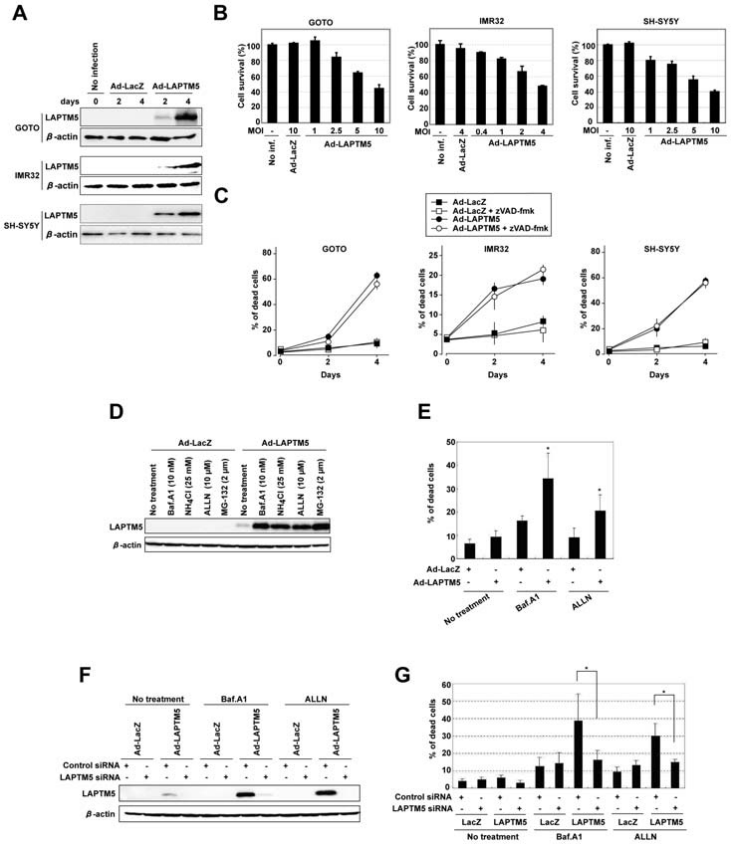
Induction of caspase-independent cell death by LAPTM5 overexpression and effect of LAPTM5 accumulation on the cell death. (A) Western blot analysis of adenovirus-infected GOTO, IMR32, and SH-SY5Y cells. The cells were infected with the *LacZ* (Ad-LacZ) or *LAPTM5* (Ad-LAPTM5) adenovirus at a MOI of 10 for GOTO and SH-SY5Y or 4 for IMR32. Two and four days after infection, whole-cell lysates was isolated, and analyzed by immunoblotting with an antibody to LAPTM5 or β-actin (internal control). Results shown are representative of two independent experiments. (B) Viability of adenovirus-infected GOTO, IMR32, and SH-SY5Y cells. GOTO (5×10^4^ cells/well), IMR32 (2×10^4^ cells/well), or SH-SY5Y (5×10^4^ cells/well) cells plated in 24-well plates were infected with Ad-LacZ or Ad-LAPTM5 at the indicated MOIs. Four days after infection, the percentage of surviving cells was determined by a colorimetric water-soluble tetrazolium salt (WST) assay. Vertical lines, SD for three experiments. No inf., no infection. (C) Frequency of dead cells among adenovirus-infected GOTO, IMR32, and SH-SY5Y cells. The cells were infected with Ad-LacZ or Ad-LAPTM5 under the same conditions as in (A) with or without treatment with the pan-caspase inhibitor zVAD-fmk at 100 µM in each cell line. Dead cells were counted 2 and 4 days after infection using the trypan blue exclusion method, and indicated as percentages. Vertical lines, SD for three experiments. (D) Western blot analysis of LAPTM5 in LAPTM5-infected GOTO cells treated with protein degradation inhibitors. Cells were infected with Ad-LacZ or Ad-LAPTM5 at a MOI of 10. One day after infection, cells were treated with Bafilomycin A1 (Baf.A1, lysosomal inhibitor), NH_4_Cl (lysosomal inhibitor), ALLN (proteasome inhibitor), or MG-132 (proteasome inhibitor) at indicated concentration for 1 day. Whole-cell lysate was analyzed by immunoblotting with indicated antibodies. The results shown represent two independent experiments. (E) Frequency of dead cells among LAPTM5-infected GOTO cells treated with protein degradation inhibitors. Cells were infected and treated with Baf.A1 or ALLN, as indicated in (D). Dead cells were counted using the trypan blue exclusion method, and indicated as percentages. Vertical lines, SD for three experiments. *t-test; *p*<0.05. (F) Western blot analysis of LAPTM5 inhibition by siRNA transfection in LAPTM5-infected GOTO cells treated with protein degradation inhibitors. Cells were transfected with control- or LAPTM5-siRNA, and infected with Ad-LacZ or -LAPTM5 at a MOI of 10. The next day cells were treated with the inhibitor Baf.A1 or ALLN for 1 day. Whole-cell lysate was analyzed by immunoblotting with indicated antibodies. The results shown represent two independent experiments. (G) Effect of siRNA transfection on frequency of dead cells among LAPTM5-infected GOTO cells treated with protein degradation inhibitors. Cells were infected and treated with Baf.A1 or ALLN, as indicated in (F). Dead cells were counted using the trypan blue exclusion method, and indicated as percentages. Vertical lines, SD for three experiments. *t-test; *p*<0.05.

To examine whether LAPTM5 contributes to the PCD of NB cells, the protein was overexpressed in NB cell lines using an adenovirus-mediated expression system. Strong expression of the LAPTM5 protein was detected at 4 days, rather than at 2 days, after infection in three cell lines, GOTO, IMR32, and SH-SY5Y (**Figure 3A**), and cell survival rates decreased in a dose-dependent manner on adenovirus-LAPTM5 (Ad-LAPTM5) infection (**Figure 3B**). Moreover, a remarkable increase in the number of dead cells with an increase in LAPTM5 protein level was observed at 4 days, after infection with Ad-LAPTM5, compared with adenovirus-LacZ (Ad-LacZ), in all cell lines. The cell death induced by Ad-LAPTM5 infection was not inhibited by treatment with 100 µM of zVAD-fmk, a pan-caspase inhibitor, although the reduction of cell viability induced by CDDP was partially inhibited by treatment with the same concentration of zVAD-fmk in SH-SY5Y cells (**Figure 3C** **and Supplementary Figure S5**). In addition, on immunostaining and western blotting, we detected almost no cleaved caspase-3 in Ad-LAPTM5-infected NB cell lines (**Supplementary Figure S5**), and found none of the typical features of apoptosis, such as nuclear fragmentation and apoptotic bodies during LAPTM5-induced cell death (data not shown). These results suggest that overexpression of LAPTM5 mainly induces a non-apoptotic cell death in a caspase-independent manner in NB cells.

Based on the immunohistochemical observations that LAPTM5 seemed to be markedly accumulated in degenerating NB cells, together with the observation *in vitro* that no cell death was detected at 2 days after infection regardless of the expression of LAPTM5, we speculated that the protein accumulated due to a decrease in turnover efficiency for degradation and its accumulation is required to induce cell death. To test this idea, we first investigated whether LAPTM5 was accumulated on treatment with inhibitors for the intracellular protein degradation system (proteasomal or lysosomal degradation). When GOTO cells at 1 day after Ad-LAPTM5 infection were treated with a proteasomal inhibitor (ALLN or MG132) or a lysosomal inhibitor (Bafilomycin A1; Baf.A1 or NH_4_Cl) for 1 day, a remarkable accumulation of LAPTM5 was detected by western blotting, compared with that in Ad-LAPTM5-infected GOTO cells without each of these treatments (**Figure 3D**). In addition, we showed that the induction of cell death was significantly enhanced in correlation with the accumulation of LAPTM5 in Ad-LAPTM5-infected GOTO cells treated with Baf.A1, compared to Ad-LAPTM5-infected GOTO cells without Baf.A1-treatment or in Ad-LacZ-infected GOTO cells treated with Baf.A1 (*p* = 0.027 or *p* = 0.042) or with ALLN, compared to Ad-LAPTM5-infected GOTO cells without treatment or Ad-LacZ-infected GOTO cells treated with ALLN (*p* = 0.022 or *p* = 0.028) (**Figure 3E**). Moreover, we examined the effect of inhibiting the accumulation of LAPTM5 with specific siRNA to confirm that the accumulation is required for the induction of cell death. When LAPTM5 siRNA-transfected GOTO cells were infected with Ad-LAPTM5, and cultured with or without Baf.A1 or ALLN, a remarkable inhibition of LAPTM5′s accumulation was confirmed by western blotting (**Figure 3F**). In this setting, the frequency of dead cells was significantly decreased in LAPTM5 siRNA-transfected GOTO cells, compared with that in control siRNA-transfected GOTO cells (*p* = 0.035 for Baf.A1; *p* = 0.022 for ALLN) (**Figure 3G**). Taken together, these results indicate that the introduced LAPTM5 is usually degraded by both proteasomal and lysosomal pathways and the accumulation of this protein is critical for the induction of cell death.

### Appearance of autophagic vacuoles during LAPTM5-induced cell death

**Figure 4:**
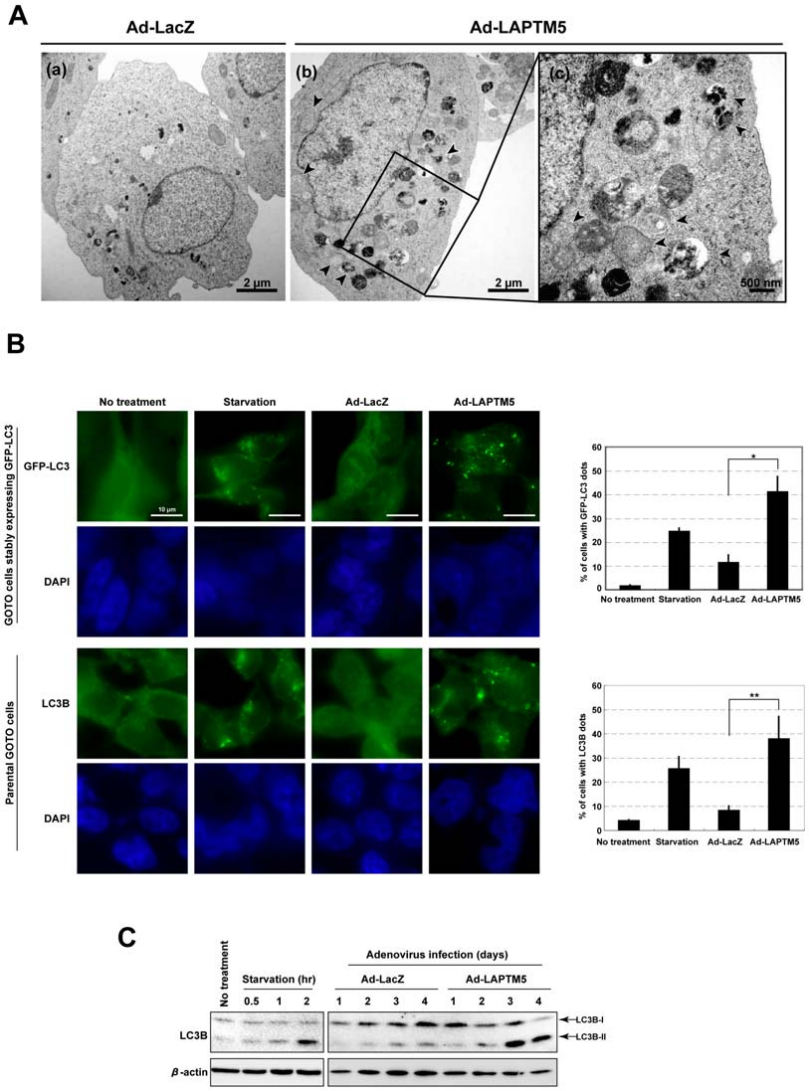
Appearance of autophagic vacuoles during LAPTM5-induced cell death. (A) Representative transmission-electron micrographs of Ad-LacZ (a) or Ad-LAPTM5 (b and c) -infected GOTO cells 4 days after infection. The (c) image was enlarged from the area enclosed by the rectangle in (b), showing the accumulation of autophagic vacuoles (arrowheads). (B) Localization of GFP-LC3 or endogenous LC3B detected by immunofluorescence microscopy. GOTO cells stably expressing GFP-LC3 (1×10^5^/well) (upper panel) or parental GOTO cells (1×10^5^/well) (lower panel) were plated on coverslips in 24-well plates, and the next day infected with Ad-LacZ or Ad-LAPTM5 at a MOI of 10. Four days later, the GOTO cells stably expressing GFP-LC3 were fixed in 4% formaldehyde, and observed under a fluorescence microscope. For parental GOTO cells, the cells were fixed in 10% TCA 4 days after infection, reacted with the LC3B antibody, and visualized with an FITC-conjugated secondary antibody. Control cells starved of nutrients were treated with EBSS for 2 hours at 5 days of plating. The frequency of cells with a punctate distribution (dot pattern) of GFP-LC3 or LC3B among all GFP-positive cells or total cells (at least 200 cells), respectively, was measured. *t-test, *p*<0.005 and **p<0.05. Vertical lines, SD for three experiments. (C) Detection of LC3B-II by western blotting. As the time-courses indicated, whole-cell lysate from GOTO cells starved of nutrients or infected with Ad-LacZ and Ad-LAPTM5 at a MOI of 10 was analyzed by immunoblotting with an antibody to endogenous LC3B or β-actin (internal control). Arrows indicate form-I or -II of LC3B. Results shown are representative of two independent experiments.

Non-apoptotic cell death in a caspase-independent manner is often accompanied by the appearance of numerous autophagic vacuoles in the cytoplasm [23], and has been proposed as one of the mechanisms for PCD during the spontaneous regression of NB tumors [12], [13]. We therefore examined whether autophagic vacuoles appear in the cytoplasm during LAPTM5-induced cell death. Electron microscopic analysis revealed numerous autophagic vacuoles in the cytoplasm of most Ad-LAPTM5-infected GOTO cells, as compared with Ad-LacZ-infected cells (**Figure 4A**). In addition, when GOTO cells stably expressing GFP-LC3 and the parental GOTO cells were infected with Ad-LAPTM5, as well as when the cells were starved to activate autophagic flux, a remarkable increase in cells showing a punctuate distribution of GFP-LC3 or endogenous LC3B was observed by immunofluorescence microscopy, compared with untreated cells or Ad-LacZ-infected cells, respectively (*p* = 0.0022 in GOTO cells stably expressing GFP-LC3, *p* = 0.0288 in parental GOTO cells) (**Figure 4B**) [23], [24]. Moreover, the cleaved form (LC3B-II) for endogenous LC3B, another indicator of the presence of autophagic vacuoles, was also clearly detected in GOTO cells during LAPTM5-induced cell death as well as starved cells by western blotting (**Figure 4C**) [23], [24]. Similar results were observed in Ad-LAPTM5-infected GOTO cells stably expressing GFP-LC3 (**Supplementary Figure S6**). In addition, the cleaved form and a punctuate distribution for endogenous LC3B were observed during LAPTM5-induced cell death in SH-SY5Y cells (**Supplementary Figure S6**). These findings indicate that overexpression of LAPTM5 induces cell death with the appearance of autophagic vacuoles in NB cells.

### Appearance of autophagic vacuoles during LAPTM5-induced cell death is attributed to an interruption of autophagic flux

**Figure 5:**
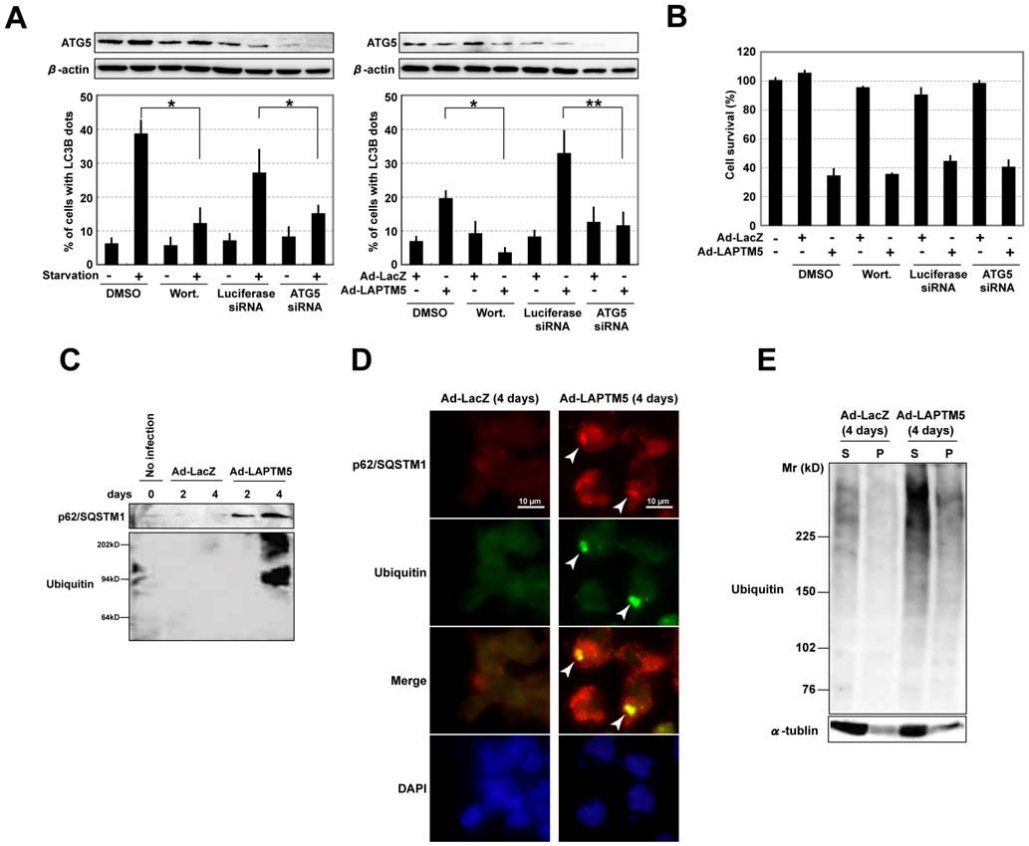
Interruption of autophagic flux during LAPTM5-induced cell death. (A) Effect of treatment with wortmannin or transfection with ATG5 siRNA in GOTO cells with a punctate distribution of LC3B. Cells (1×10^5^/well) were plated on coverslips in 24-well plates and the next day treated with wortmannin (Wort.; 200 nM) or DMSO (0.02%), or transfected with ATG5 siRNA (20 nM) or Luciferase siRNA (20 nM). *Left*; 4 days after the treatment or transfection, cells were treated with EBSS for 2 hours. *Right*; the plated cells were infected with Ad-LacZ or Ad-LAPTM5 at a MOI of 10 for 4 days with treatment with DMSO or wortmannin, or with transfection with Luciferase or ATG5 siRNA. Then, cells were fixed in 10% TCA, reacted with the LC3B antibody, and visualized with an FITC-conjugated secondary antibody. *Upper*; western blotting analysis of ATG5. Whole-cell lysate was analyzed by immunoblotting with an antibody to ATG5 or β-actin (internal control). *Lower*; frequency of cells showing a punctate distribution of LC3B among cells (at least 200 cells) was measured. *t-test, *p*<0.001 and **p<0.01. Vertical lines, SD for three experiments. (B) Effect of treatment with wortmannin or the knockdown of ATG5 during LAPTM5-induced cell death. GOTO cells (1×10^4^/well) were plated in 96-well plates and infected with Ad-LacZ or LAPTM5 at a MOI of 10 with treatment with DMSO or wortmannin (Wort.), or with transfection with Luciferase or ATG5 siRNA. Four days after infection, numbers of viable cells were assessed by WST assay. Vertical lines, SD for three experiments. (C) Western blot analysis of p62/SQSTM1 and ubiquitin in LAPTM5-infected GOTO cells. The same whole-cell lysates indicated in Figure 3A were analyzed by immunoblotting with indicated antibodies. The results shown represent two independent experiments. (D) Immunofluorescence microscopy for p62/SQSTM1 and ubiquitin in LAPTM5-infected GOTO cells. *Upper*; GOTO cells (1×10^5^/well) were plated on coverslips in 24-well plates, and infected with Ad-LAPTM5 at a MOI of 10. Four days after infection, the cells were fixed in 4% formaldehyde, reacted with p62/SQSTM1 and ubiquitin antibodies, and visualized with an FITC- or Alexa Fluor 594-conjugated secondary antibody. Arrowheads indicate the cells with p62/SQSTM1-ubiquitin positive inclusion-like body. DAPI was used for counterstaining. (E) Western blotting of ubiquitinated proteins. Four days after infection, GOTO cells were lysed in a 0.1% Triton-X solution. The Triton-insoluble pellet (P) was lysed in a 2% SDS buffer. The Triton-X soluble (S) or -insoluble (P) lysate was loaded on a 6% SDS-PAGE gel and analyzed by immunoblotting with an antibody to ubiquitin or α-tublin. For both soluble and insoluble samples, ubiquitinated proteins were found to be accumulated in Ad-LAPTM5-infected GOTO cells, compared with Ad-LacZ-infected GOTO cells. The results shown represent two independent experiments.

The autophagic process functions as a flux, with portions of the cytosol and intracellular organelles sequestered into the autophagosome. The autophagosome fuses with intracellular vesicles and finally matures into the autolysosome through fusion with the lysosome, which supplies acid hydrolases, for bulk degradation. While the autophagic process is rapidly activated in the absence of nutrients for cell survival, the process also occurs constitutively at a low level in cells (basal autophagic flux) [23], [24]. Importantly, the appearance of numerous autophagic vacuoles during cell death does not necessarily reflect “so-called autophagic cell death” attributed to activation of the autophagic process [23]. When the formation of autophagosomes was inhibited by treatment with wortmannin, an autophagic inhibitor, or by the knockdown of ATG5 expression during cultivation in a starved state or LAPTM5-induced cell death in GOTO cells, the number of cells showing a punctate pattern of LC3B expression was significantly decreased (**Figure 5A** and **Supplementary Figure S7**). Even though the autophagic process was experimentally inhibited, the frequency of LAPTM5-induced cell death was not affected (**Figure 5B**), indicating that LAPTM5-induced cell death is not “so-called autophagic cell death” [23] and the appearance of autophagic vacuoles during LAPTM5-induced cell death may result in the accumulation of immature autophagic vacuoles due to an interruption of basal autophagic flux.

Accumulating evidence suggests that p62/SQSTM1 and some ubiquitinated proteins are degraded during the autophagic process by which p62/SQSTM1 recruits ubiquitinated proteins into the autophagosome through interaction directly with LC3 protein to be degraded by autophagy [24]–[26]. In addition, the genetic disruption of autophagic flux in mouse neurons led to the accumulation of p62/SQSTM1 and ubiquitinated proteins and the formation of inclusion bodies with these proteins, resulting in a loss of neurons by cell death [27], [28]. To further confirm that basal autophagic flux is interrupted during LAPTM5-induced cell death, we examined levels of p62/SQSTM1 protein and ubiquitinated proteins. As expected, a remarkable accumulation of both p62/SQSTM and ubiquitinated proteins was observed on western blotting (**Figure 5C**) and immunofluorescence analysis (**Supplementary Figure S8**). These proteins were frequently co-localized as inclusion-like bodies (**Figure 5D**) and ubiquitinated protein levels were increased in the Triton-X insoluble fraction, not only the soluble fraction, in Ad-LAPTM5-infected GOTO cells (**Figure 5E**). We also confirmed that p62/SQSTM and GFP-LC3 were occasionally co-localized to cytoplasmic spots (or ring-like structures) as autophagosomes in Ad-LAPTM5-infected GOTO cells (data not shown). These findings strongly suggest that overexpression of LAPTM5 leads to an interruption of basal autophagic flux, and results in the accumulation of p62/SQSTM1 and ubiqutinated proteins, as well as of immature autophagic vacuoles, that is, “cell death with impaired autophagy” [23].

### Accumulation of LAPTM5-positive vesicles during LAPTM5-induced cell death

**Figure 6:**
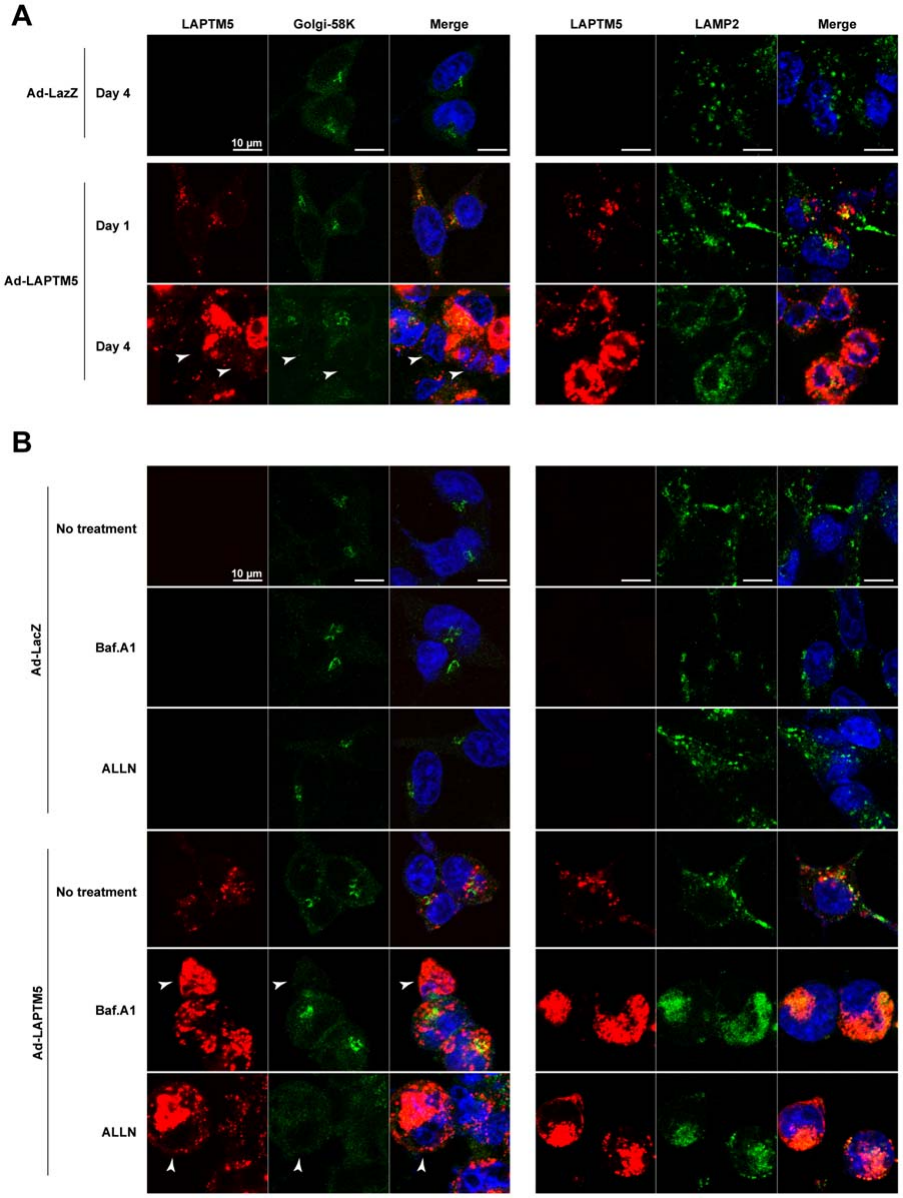
Localization of accumulated LAPTM5 during LAPTM5-induced cell death. (A) Representative image of the co-staining of Golgi-58K or LAMP2 (Green) and LAPTM5 (Red). GOTO cells (1×10^5^/well) were plated on coverslips in 24-well plates and the next day infected with Ad-LacZ or Ad-LAPTM5 at a MOI of 10. One or four days after infection, the cells were fixed in 10% TCA, reacted with Golgi-58K or LAMP2 and LAPTM5 antibodies, and visualized with FITC- or Alexa Fluor 594-conjugated secondary antibodies. DAPI was used for counterstaining. Arrowheads indicate the cells with a loss of Golgi apparatus structures. (B) Representative image of the co-staining of Golgi-58K or LAMP2 (Green) and LAPTM5 (Red). GOTO cells were infected with Ad-LacZ or Ad-LAPTM5 a MOI of 10, and next day treated with Bafilomycin A1 (Baf.A1) or ALLN for 1 day. Arrowheads indicate the cells with a loss of Golgi apparatus structures. Then immunofluorecent images were obtained as in (A).

LAPTM5 is a multispanning transmembrane protein that resides in lysosomes [29]. Further, it has been demonstrated that LAPTM5 contributes to vesicle trafficking from the Golgi apparatus to the lysosome [30]. Therefore, we investigated the subcellular distribution of LAPTM5 in cells together with that of Golgi-58K (Golgi marker) or LAMP2 (Lysosome marker) by conforcal microscopy. As shown in **Figure 6A**, the exogenously expressed LAPTM5 was localized to vesicles and exhibited a punctuate pattern, and the LAPTM5-positive vesicles did not colocalize with the Golgi apparatus, but partially colocalized with LAMP2-positive lysosomes 1 day after the infection with Ad-LAPTM5. Four days after the infection, LAPTM5-positive vesicles were remarkably accumulated as larger entities in dying cells and colocalized with neither the Golgi apparatus nor LAMP2-positive lysosomes. Rather, Golgi-58K-positive structures and LAPM-2-positive punctuate pattern were shown to be reduced in dying cells with a strong accumulation of LAPTM5-positive vesicles.

Since treatment with lysosomal or proteasomal-degradation inhibitors significantly enhanced LAPTM5-induced cell death combined with the accumulation of exogenously expressed LAPTM5 (**Figure 3D and 3E**), we next examined the subcellular localization of accumulated LAPTM5 under such conditions. LAPTM5 protein was remarkably accumulated as large vesicles in Ad-LAPTM5-infected GOTO cells treated with 10 nM Baf.A1 or 10 µM ALLN (**Figure 6B**) for one day, similar to the pattern observed 4 days after the infection with Ad-LAPTM5 (**Figure 6A**). In addition, a reduction in Golgi-58K-positive structure and LAPM-2-positive punctuate pattern was also observed in dying cells with a strong accumulation of LAPTM5-positive vesicles. Alternatively, when GOTO cells were infected with Ad-LAPTM5 for 2 days under mild treatment with 2 nM of Baf.A1 or 2 uM of ALLN, LAPTM5-positive vesicles were frequently co-localized with LAMP2-positive lysosomes (**Supplementary Figure S9**). Based on the these findings, it is considered that LAPTM5 intrinsically localizes to trafficking vesicles from Golgi to lysosomes in NB cells, and an increase of LAPTM5 protein leads to the production of LAPTM5-positive vesicles. Further the gradual accumulation of these vesicles by consecutive overexpression of LAPTM5 and/or a treatment with lysosomal or proteasomal inhibitor leads to losses of the Golgi apparatus and punctate pattern of lysosomes. Consequently, the accumulation of LAPTM5-positive vesicles might lead to lysosomal destabilization.

### Induction of lysosomal destabilization with lysosomal-membrane permeabilization (LMP) during LAPTM5-induced cell death

**Figure 7:**
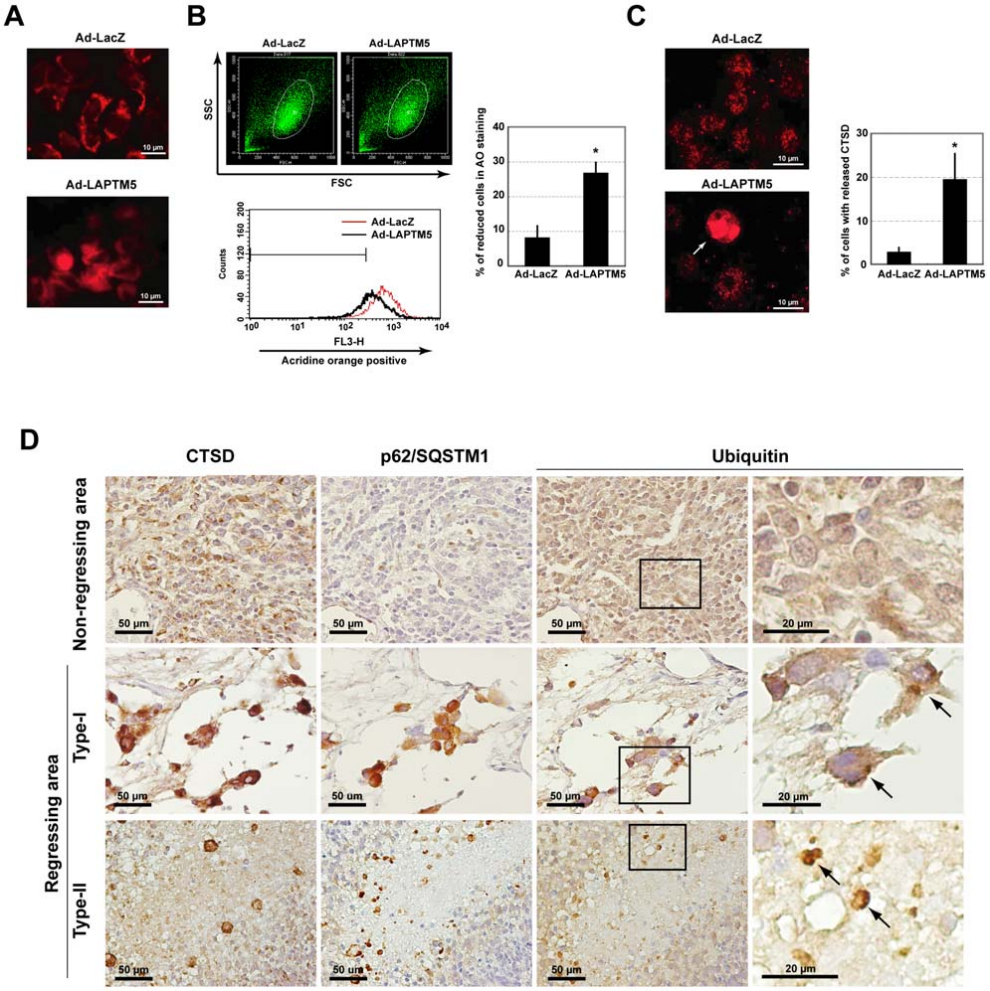
Induction of lysosomal-membrane permeabilization (LMP) during LAPTM5-induced cell death. (A) Representative images of LysoTracker Rhodamine (LTR) staining in infected GOTO cells. GOTO cells (1×10^5^/well) were plated on coverslips in 24-well plates, and infected with Ad-LAPTM5 at a MOI of 10. Four days after infection, cells were stained with LTR (100 nM) for 30 min at 37°C, washed twice and fixed in 4% formaldehyde, and observed by fluorescence microscopy. (B) Acridine orange (AO) uptake analysis in infected GOTO cells. GOTO cells (2×10^5^/well) were plated in 6 well plates, and infected with Ad-LAPTM5 at a MOI of 10. Four days later, cells were stained with AO (5 µg/ml, 30 min). Detached cells were removed, and attached cells were collected by trypsinization and washed twice with PBS. *Left*-*upper*; scatter blots of FSC and SSC for Ad-LacZ or Ad-LAPTM5-infected cells. The main population of cells was gated and analyzed by FACS. *Left*-*lower*; the intensity of staining was measured by FACS using a channel of FL3; the range containing >99% of the cells without AO fluorescence was gated. Black line indicates Ad-LAPTM5-infected cells; red line, Ad-LacZ-infected cells. The frequency of cells with a reduction in AO staining was increased among Ad-LAPTM5-infected GOTO cells. *Right*; percentage of cells with a reduction in AO fluorescence. Vertical lines, SD for four separate experiments. *t-test, *p*<0.01. (C) Representative images of staining for cathepsin D (CTSD) in infected GOTO cells. GOTO cells (1×10^5^/well) were plated on coverslips in 24-well plates, and the next day infected with Ad-LacZ or Ad-LAPTM5 at a MOI of 10. Four days after infection, the cells were fixed in 10% TCA, reacted with a CTSD antibody, and visualized with an Alexa Fluo 594-conjugated secondary antibody. The percentage of cells that released CTSD (arrow) was measured. Vertical lines, SD for three separate experiments. *t-test, *p*<0.05. (D) Representative images of staining for CTSD, p62/SQSTM1, or ubiquitin in non-regressing or regressing areas within favorable NB tumors. Serial tumor sections were stained with each antibody. The area enclosed by the rectangle is enlarged at the right. Arrows indicate cells with ubiquitin-positive inclusion bodies.

Based on the results of the immunofluorescence analysis (**Figure 6**), we examined whether lysosomal destabilization occurs during LAPTM5-induced cell death. Along with the loss of a punctuate pattern of LysoTracker Rhodamine (LTR) staining (Pale cells) which reflects lysosomal destabilization via lysosomal-membrane permeabilization (LMP) [31]–[35], we observed the loss of the punctuate pattern in proportion to an increase in cell death among LAPTM5-infected GOTO cells (2.7-fold in GOTO, *p* = 0.0007, compared with Ad-LacZ; **Figure 7A** and **Supplementary Figure S10**). In addition, we found a diminution in acridine orange (AO) uptake [31], another indicator for LMP, during LAPTM5-induced cell death, in a FACS analysis (*p* = 0.0001 in Ad-LAPTM5-infected GOTO cells compared to those infected with Ad-LacZ; **Figure 7B**). Since lysosomal destabilization with LMP leads to the leakage of enzymes from the lysosome into the cytosol, we examined the subcellular distribution of cathepsin D (CTSD), a lysosomal enzyme [32]. The number of cells leaking CTSD into the cytosol was significantly increased among Ad-LAPTM5-infected GOTO cells compared with Ad-LacZ-infected GOTO cells (*p* = 0.0388) (**Figure 7C**). In addition, we also observed that the fluorescein isothiocyanate-labelled dextran (FITC-dextran; 40kD), which was incorporated intact lysosomes, also was leaked into the cytosol (**Supplementary Figure S10** and **Supplementary Methods S1**) [31]. Moreover, treatment with ciprofloxacin (CPX), a known inducer of LMP [30], also resulted in the presence of the GFP-LC3-II form and the accumulation of p62/SQSTM1 proteins (**Supplementary Figure S11**). Taken together, these findings suggest the accumulation of LAPTM5 in NB cells to cause lysosomal cell death due to lysosomal destabilization with LMP. The lysosomal dysfunction caused by LAPTM5-mediated LMP might lead to an interruption to basal autophagy flux, probably resulting in the increased number of immature autophagic vacuoles.

To confirm whether levels of CTSD, p62/SQSTM1, and ubiqutinated proteins are increased in degenerating NB cells as observed *in vitro*, we performed an immunohistochemical analysis using each specific antibody in primary NB tumors. As shown in **Figure 7D**, levels for each of these proteins were remarkably increased in degenerating NB cells within regressing areas, compared with levels in non-degenerating NB cells, in the mass-screened NB tumors. Notably, ubiquitin-positive inclusion bodies were occasionally observed in degenerating NB cells. In addition, the CTSD protein appeared to fill the cytoplasm. These observations suggest that degenerating NB cells accumulating LAPTM5 protein undergo lysosomal cell death with LMP and impaired autophagy, and this process is closely associated with the PCD of NB cells during the spontaneous regression of favorable NB tumors.

## Discussion

In the present study, the BAMCA method revealed that *LAPTM5* was highly methylated and its expression was transcriptionally down-regulated in NB cell lines. Unexpectedly, the methylation/down-regulation of this gene was detected in all the primary NB tumors and cell lines examined, and we further could detect no difference in methylation status and expression level between favorable and unfavorable NB tumors. Based on our findings that the mRNA level of *LAPTM5* restored after treatment with 5-aza-dC was still lower than in normal adrenal glands and the frequency with which *LAPTM5* was methylated was decreased in benign GNs, we conclude that the methylation of *LAPTM5* in NB cells is not aberrant methylation for an irreversible shut-off of the expression as occurs in CpG islands of classical tumor suppressor genes (indeed *LAPTM5* has no CpG islands) and may contribute to maintaining a constitutively down-regulated state and/or prevent leaking of the expression of this gene under conditions without transcriptional activation in an NB cell lineage-specific manner. Indeed, a recent report also found a decrease in the methylation of *LAPTM5* during normal lung development and in correlation with the differentiation state of lung tumors [36].

**Figure 8:**
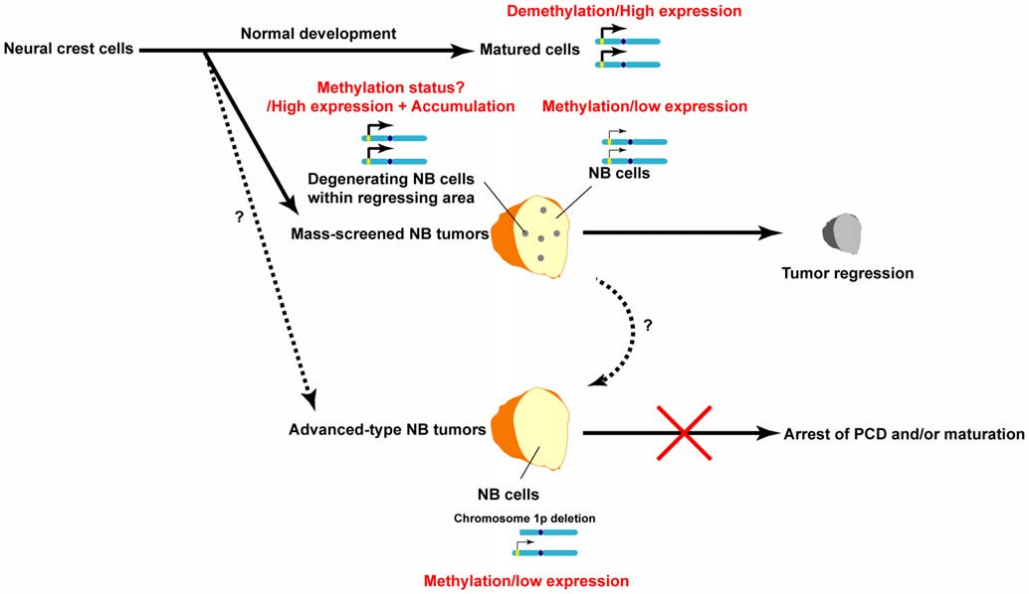
Model of the relationship between LAPTM5-mediated degeneration and propensity for spontaneous regression of NB tumors.

One key finding of the present study is that LAPTM5-mediated programmed cell death (PCD) was closely associated with the spontaneous regression of mass-screened NB tumors. As illustrated in **Figure 8**, although *LAPTM5* expression usually appears to be down-regulated through DNA methylation in both favorable and unfavorable NBs, it is up-regulated in degenerating NB cells within locally limited regressing areas of favorable NBs. Our immunohistochemical analyses revealed that LAPTM5-positive degenerating NB cells were frequently detected in mass-screened NB tumors, but not in clinically detected unfavorable tumors, establishing a clear correlation between LAPTM5-mediated PCD and the propensity of NB tumors to undergo regression. The mass-screened NB tumors contained LAPTM5-positive degenerating NB cells, nonetheless we could not detect a significant difference in the mRNA expression of this gene between favorable and unfavorable tumor samples, suggesting the up-regulation of LAPTM5 expression to be involved in the pro-death role in the late phase of PCD in regressing tumors and LAPTM5-positive degenerating NB cells are a small proportion in mass-screened NB tumors.

Our observations also revealed that the LAPTM5 protein to be more significantly accumulated in degenerating NB cells than differentiating NB cells and the accumulation to be required to induce cell death in NB cells. Our *in vitro* study showed that the consecutive overexpression of LAPTM5 led to the accumulation of LAPTM5-positive vesicles and the accumulation was enhanced by treatment with inhibitors for either the lysosomal or proteasomal pathway. Moreover, under cellular stress caused by treatment with a neurotoxin (such as 1-methyl-4-phenylpyridinium; MPP^+^) or H_2_O_2_, or on cell differentiation induced by treatment with retinoic acid (RA), we found a remarkable increase of LAPTM5 expression at the transcriptional level. In addition, we showed an accumulation of LAPTM5 protein level in RA-induced differentiated IMR32 cells treated with Baf.A1 or ALLN (**Supplementary Figure S12** and **Supplementary Methods S1**). Thus, the results suggest that lysosomal or proteosomal degradation of LAPTM5, whose impairment might enhance the accumulation of the protein and/or LAPTM5-positve vesicles, in addition to the consecutive transcriptional activation of *LAPTM5* by cellular stress, also plays a critical role in degeneration and cell death in NB cells. Indeed, the spontaneous regression of clinically favorable NB tumors is known to occur over a period of several months [4], [14]–[17].

Advanced-type NB tumors are presumed to have a very low chance of regressing, and the final outcome for any NB tumor is likely to be attributable to cytogenetic aberrations as well as biological differences [1]–[3]. Deletion of the short arm of chromosome 1 (1p), observed in about 40% of NBs, is strongly associated with a poor prognosis, and highly associated with *MYCN* amplification, which is itself a strong indicator of a poor outcome for advanced-type NB tumors [1]–[3], [37]. Accumulating evidence suggests that multiple candidates, including *p73*, *CDH5*, and *KIF1β*, for genes having a NB-suppressive role within the 1p36-critical region [1]–[3], [38]–[40]. In addition, some of the 1p deletions in *MYCN*-amplified NB tumors are quite large, involving a proximal segment spanning 1p32–p36; tumors having this extensive loss of 1p make up a subset of advanced-type NBs with a poorer prognosis [41], [42]. *LAPTM5* is mapped at 1p35, not at 1p36, and the copy-number of the *LAPTM5* gene locus is also frequently decreased in NB cell lines (**Supplementary Figure S2**). Therefore, the decrease of *LAPTM5* gene dosage may also act to prevent accumulation of LAPTM5-positive vesicles and LAPTM5-mediated PCD in advanced-type NB tumors with a large deletion of 1p.

A second key finding is that the cell death mediated by the accumulation of LAPTM5 protein in NB cells is a lysosomal cell death with the typical features of lysosomal destabilization through LMP and with the impaired autophagy in a caspase-independent manner, as a molecular mechanism for spontaneous regression of NB tumors. At this time, it is not clear how the accumulated LAPTM5 contributes to the lysosomal destabilization in degenerating NB cells and how the non-accumulated LAPTM5 functions in differentiating NB cells, adrenal medulla cells, or ganglion cells in GNs. A recent report demonstrated that LAPTM5 plays an important role in the lysosomal degradation of specific substrates in T cells [43]. Therefore, in “differentiating NB cells” or “matured neuroblastic cells” as well as in T cells, LAPTM5 may also function in the lysosomal degradation system for specific substrate(s). Our *in vitro* study showed that LAPTM5 intrinsically localizes to trafficking vesicles from the Golgi apparatus to the lysosomes in NB cells, and consecutive overexpression of LAPTM5 leads to the accumulation of LAPTM5-positive vesicles together with losses of the Golgi apparatus and punctate pattern of lysosomes. It has been reported that LAPTM5 contributes to vesicle trafficking from the Golgi apparatus to lysosome [30]. In addition, lysosomal membrane proteins such as LAMP1 and LAMP2 were transported from the Golgi apparatus to the lysosomes [44]. Therefore, the accumulation of LAPTM5-positive vesicles may lead to a disruption of intracellular vesicle trafficking in the Golgi-lysosome pathway, resulting in lysosomal destabilization, accompanied by interruption of provision of lysosomal contents into the lysosomes, in “degenerating NB cells”. Interestingly, a recent report showed that a down-regulation of lysosomal membrane proteins, LAMP1 and LAMP2, increased sensitivity to the lysosomal cell death and those accumulation induced by inhibition of lysosomal proteases contribute to the resistance to lysosomal cell death [45].

On the other hand, our study also revealed that the lysosomal disruption by treatment with lysosomal inhibitor led to the accumulation for these vesicles (by a decrease of removal in the lysosomes), although LAPTM5-positive vesicles were usually transported into the lysosomes, and to be degraded. A dysfunction of proteasomal protein degradation also led to the accumulation of LAPTM5-positive vesicles. Thus, this protein may be also degraded by ubiquitin-proteasome system to regulate the production of LAPTM5-positive vesicles. Indeed, LAPTM5 protein can be ubiquitinated by NEDD4, an E3 ubiquitin ligase [30].

This cell death is also characterized by the accumulation of autophagic vacuoles and ubiquitinated proteins and the occasional formation of ubiquitin-positive inclusion bodies as occurs in neurodegenerative diseases (NDs) [46], suggesting that a common molecular mechanism underlies the degeneration of cells in the regression of NB tumors and in NDs. We consider the ND-like features of degenerating NB cells attributable to an interruption of autophagic flux caused by a loss of intact lysosomes through LAPTM5-mediated lysosomal destabilization, although we can not exclude the existence of some other mechanism, given results of studies where autophagic flux or lysosomal degradation were genetically disrupted in mice [27], [28].

Finally, it has been reported that H-Ras is also highly expressed in degenerating NB cells and its overexpression can induce caspase-independent cell death with autophagic vacuoles in NB cells [12]. However, it has been not clarified whether this cell death is attributable to an interruption of autophagic flux. Clarification of the relationship between H-Ras and LAPTM5 in signal transduction will require further examination.

## Methods

### Cell lines and tumor samples

All 10 human NB cell lines used (GOTO, IMR32, SJ-N-CG, CHP134, MP-N-MS, KP-N-RT, SK-N-AS, SH-SY5Y, SK-N-SH, and SK-N-KP) had been established from surgically restricted tumors, and maintained as described elsewhere. Primary samples were obtained during surgery from NB patients undergoing tumor resection at University Hospital, Kyoto Prefectural University of Medicine from 1986 to 2003, with written consent by the parents of each patient in the formal style and after approval by the local ethics committee. Staging was evaluated according to the criteria of the International Staging System (INSS) [20]. Patients were treated according to previously described protocols [20]. Tumor samples were frozen immediately and stored at −80°C until required.

### Cell culture and treatments

All cell lines were grown in RPMI1640 medium supplemented with 10% FCS, penicillin, and streptomycin. Earle's Balanced Salt Solution (EBSS) was from Invitrogen. For treatment with drugs in cell cultures, wortmannin (Sigma), zVAD-fmk (Peptide Institute), Bafilomycin A1 (Sigma), ALLN (Calbiochem), NH_4_Cl (Wako), and MG-132 (Calbiochem) were used.

### Generation of specific antibody for LAPTM5

An anti-LAPTM5 polyclonal antibody against a peptide, CEEALSLPSKTPEGG, from the carboxyl terminus was generated in rabbits (Qiagen), and affinity-purified.

### DNA methylation analysis

For bisulfite sequencing, genomic DNA was treated with sodium bisulfite and amplified with primer sets for sequences of interest. PCR products were sub-cloned and sequenced.

To investigate methylation status in primary tumor samples, methylation-sensitive single nucleotide primer extension (Ms-SNuPE) was performed [47]. Genomic regions containing two CG sites were amplified using sodium bisulfite-treated DNA; the PCR products were purified from agarose gels and used as templates for analyzing methylation using Ms-SNuPE primers specific for each site. The MS-SNuPE products were run on 15% polyacrylamide gels, and the resulting signals were quantified with a Phosphoimager analysis system (Molecular Dynamics). Methylation values were calculated based on results from two CG sites, according to the following equation: [methylated *C*/(methylated *C* + unmethylated *T*)]×100. Primer sequences used in the analysis are provided in **Supplementary Table S3**.

### Reverse Transcription (RT)-PCR

Single-stranded cDNA generated from total RNA [20] was amplified with a primer set specific for the gene being examined. Real-time quantitative PCR experiments were done on an ABI-7900 with TaqMan probes (Applied Biosystems). The gene encoding glyceraldehyde-3-phosphate dehydrogenase (*GAPDH*) served as an endogenous control. Each sample was normalized on the basis of its *GAPDH* content. Primer sequences are provided in **Supplementary Table S3**.

### Immunohistochemistry

Sections from paraffin-embedded tumor samples were deparaffinized by xylene, and rehydrated in ethanol. After the retrieval of antigens by boiling in 10 mM citrate buffer (pH 6.0), the sections were treated with 0.3% hydrogen peroxide in methanol to inactivate the endogenous peroxidase. Then, the sections were incubated with an antibody against LAPTM5 (1∶200), cleaved caspase-3 (1∶200, Cell signaling), CD20 (BCA-B20; 1∶10, kindly provided by Dr H. Tsuda), p62/SQSTM1 (1∶200, Santa Cruz Biotechnology), ubiquitin (1∶200, Dako), or cathepsin D (1∶200, Chemicom) for 1 hour at room temperature. The bound antibody was visualized using diaminobenzidine as a chromogen (VECTASTAIN^_^ Elute ABC kit, Vector Laboratories), and the sections were lightly counterstained with hematoxylin. The specificity of LAPTM5 immunostaining was confirmed by omission of the primary antibody, using rabbit preimmune-serum as the primary antibody, or by the immunoabsorption method.

### Western blotting

Whole-cell extracts were prepared in RIPA buffer containing a proteinase inhibitor cocktail (Roche). Western blotting was performed as described previously [20]. Antibodies against LAPTM5 (1∶1000), MYCN (1∶500, Santa Cruz Biotechnology), p62/SQSTM1 (1∶1000), ubiquitin (1∶1000), ATG5 (1∶500, Abgent), LC3B (1∶4000, Sigma), β-actin (1∶4000, Sigma), and GFP (1∶1000, MBL) were used according to the manufacturer's instructions. As an internal control for western blotting, the blots were stripped and reprobed with a monoclonal antibody against β-actin (Sigma).

### Isolation of cells stably expressing GFP-LC3

The GFP-LC3 expression vector was kindly provided by Dr T. Yoshimori. Cells were transfected with GFP-LC3 using Lipofectoamine 2000 (Invitrogen) according to instructions. Then they were cultured in the presence of G418 (Sigma) for 3 weeks and the resistant colonies pooled in a mass were used as the cells stably expressing GFP-LC3.

### Immunofluorescence microscopy

The cells were fixed in 4% formaldehyde or 10% trichloroacetic acid (TCA), permeabilized with 0.2% Triton X-100 and treated with blocking solution (1% BSA/0.01% Triton X-100 in PBS), and then incubated at 37°C with the primary antibody for 3 hours or overnight. The bound antibody was visualized using a FITC-conjugated or Alexa Fluor 594-conjugated secondary antibody. After mounting with DAPI (4′,6′-diamidino-2-phenylindole) to stain nuclei, the cells were observed under a fluorescence microscope (ECLIPSE 800; Nikon) or a conforcal microscopy (Carl Zeiss). Antibodies against LAPTM5 (1∶200), LC3B (1∶1000), p62/SQSTM1 (1∶200), ubiquitin (1∶200), Golgi-58K (1∶200, Sigma), and cathepsin D (1∶200) were used according to the manufacturers' recommendations.

### RNA interference

The siRNAs for LAPTM5 (siGENOME SMARTpool M-019880) and for Control (siControl, Non-targeting siRNA #2) were from Dharmacon. The siRNAs for Luciferase (CGUACGCGGAAUACUUCGA) and for ATG5 (GCAACUCUGGAUGGGAUUG) were from Sigma. The siRNAs were transfected into cells using Lipofectoamine RNAi MAX (Invitrogen) according to the manufacturer's directions and after 4–6 hours, the medium was renewed.

### Recombinant Adenovirus

The replication-defective recombinant adenovirus was constructed with the Adenovirus Expression Vector Kit (Takara) following the manufacturer's recommendations. Cosmid vector-inserted full-length *LAPTM5* cDNA (pAxCAwtit-LAPTM5) was generated and the adenovirus was propagated in HEK293 cells, and stored at −80°C prior to use. As a control, an Ad-LacZ adenovirus encoding the β-galactosidase gene was constructed from the cosmid pAxCAi*LacZ* (TaKaRa). Viral titers were measured in pfu/ml by a limiting-dilution method using the HEK293 cells. Cells were infected with each MOI (multiplicity of infection; PFU/cell). Primer sequences for the construction of vectors are provided in **Supplementary Table S3**.

### Cell death and survival assays

For the cell death assay, numbers of dead cells were determined using the trypan blue exclusion method. A minimum of 100 cells were counted in each experiment. For cell survival assay, numbers of viable cells were assessed by a colorimetric water-soluble tetrazolium salt assay (WST assay, cell counting kit-8; Dojindo Laboratories).

### Electron microscopy

The cells were fixed with 2.5% glutaraldehyde in 0.1 M phosphate-buffered saline (PBS) overnight. They were washed with 0.1 M PBS at 4°C and post-fixed with 1% O_S_O_4_ buffered in 0.1 M PBS for 2 h. Then the cells were dehydrated in a graded series of ethanol solutions and embedded in Epon 812. Ultrathin (90 nm) sections were collected on copper grids, double-stained with uranyl acetate and lead citrate, and examined by transmission electron microscopy (H-7100, Hitachi).

### LysoTracker Rhodamine (LTR) staining and Acridine orange (AO) uptake analysis

For LTR staining, the cells were stained with 100 nM of LTR (Molecular Probes) for 30 min at 37°C. After two washes with PBS, the cells were analyzed by fluorescence microscopy for LTR. For AO uptake analysis, the cells were stained with 5 µg/ml of AO (Sigma) for 30 min at 37°C. Then, detached cells were removed, attached cells were collected by tripsinization and washed twice with PBS. The cells untaken AO were detected by flow cytometry using a FACScan (Becton-Dickinson).

### Statistical analysis

The results of quantitative *in vitro* analyses are presented as the mean and SD. Differences were compared with a two-sided test (Student's t-test).

## Supporting Information

Methods S1 Supplementary methods and Supplementary references
(0.03 MB DOC)

Table S1 List of BACs containing possibly methylated regions in NB cell lines compared to PBMNCs by BAMCA analysis a; Distance from the top of short arm on chromosome 1. b; BAMCA ratios were indicated by resulting for duplicate spots (1 and 2). c; The presence (“yes”) or absence (“no”) of expression for each gene was determined by RT-PCR as indicated in Supplementary Figure S1. d; The presence (“yes”) or absence (“no”) of differential methylation in SmaI sites for each gene was determined by MS-PCR as indicated in supplementary Figure S1. e; The methylation status of LAPTM5 in primary NB tumors was determined by COBRA as indicated in supplementary Figure S1. “-” indicates “not tested”.
(0.02 MB DOC)

Table S2 Summary of copy-number aberrations for MYCN, LAPTM5, and 1p35 region in 13 NB cell lines a; Copy-number status for MYCN gene locus and 1p35 region was determined by array-CGH and conventional CGH analysis. “yes” indicates the presence of MYCN amplification or 1p35 loss, and “no” indicates the absence of them. b; Two probes were used for FISH analysis. 418B22 (BAC clone), spotted on 1p36-contig array, is mapped on 1p35 and contain LAPTM5 gene locus. The pUC1.77 (plasmid) as a control is mapped on the pericentromeric region of chromosome 1.
(0.02 MB DOC)

Table S3 Primer sequences
(0.02 MB DOC)

Figure S1 Validation of candidate genes down-regulated through DNA methylation (A) Schematic diagram of BAMCA experiments. DNA fragments generated by the MCA method from NB cell lines (Green) and from the references (Red) were labeled with Cy3 or Cy5 respectively, and co-hybridized on the in-house1p35–p36 contig array. (B) Screening of down-regulated genes by RT-PCR analysis. GAPDH served as an internal control. Adr, normal adrenal glands; S1 NBs, pooled samples from five stage-1 NB tumors. (C) Determination of methylation status of SmaI sites within 418B22, a BAC clone that includes LAPTM5, by MS-PCR in PBMNCs and the two NB cell lines GOTO and IMR32. Sixteen primer sets were used for PCR in genomic regions including at least two SmaI sites in a distance range of 200–2,000 bp, because the MCA procedure produced a genomic fragment having two adjacent methylated SmaI sites at each end. Black and white circles indicate the presence (methylated) or absence (unmethylated) of PCR products, respectively. The region (including no. 12–16) shown by a gray box indicates the NB-specific methylated region at the LAPTM5 locus. (D) Determination of methylation status at CG sites around the transcriptional-start site of the LAPTM5 gene by bisulfite sequencing. A total of 32 CG sites within 1 kb of the start site were analyzed using two primer sets (regions-I and -II). An arrow at the start-site shows the direction of transcription. Arrowheads indicate two SmaI sites that located within the PCR product produced by no. 14 primers, as indicated in (C). GOTO and IMR32 cells were both widely methylated, whereas PBMNCs and an EBV-transformed lymphocyte cell line (LCL) were almost completely unmethylated. (E) Representative images of COBRA for LAPTM5 in primary NB tumors. Methylation of this gene was detected in regions-I and -II in all NB tumors examined. Bisulfite-PCR products for each of the two regions indicated in (D) were digested with HinfI (region-I) and TaqI (region-II). Arrows indicate where bands would show the presence of methylation.
(0.85 MB TIF)

Figure S2 Analysis of DNA copy-number aberrations in the 1p region in NB cell lines Frequency of 1p loss in NB cell lines revealed by array-CGH. In house ‘MCG cancer array-800’-spotted arbitrary BACs, including 43 BACs on 1p, were used for analyzing 13 NB cell lines (GOTO, IMR32, SJ-N-CG, CHP134, MP-N-MS, KP-N-RT, SMS-KCN, SK-N-DZ, SMS-KAN, SK-N-AS, SH-SY5Y, SK-N-SH, and SK-N-KP). The sideways red (4 non-amplified NB cell lines) or blue (MYCN-amplified NB cell lines) bars indicate frequencies of loss on each BAC, respectively. A large range of loss on 1p, including the LAPTM5 locus, was detected frequently in MYCN-amplified NB cell lines.
(0.93 MB TIF)

Figure S3 Methylation analysis in primary NB tumors and ganglioneuromas (GNs) by the methylation-sensitive single-nucleotide primer extension (Ms-SNuPE) method (A) Positions of primers used in the Ms-SNuPE analysis. Bisulfite-PCR products for region-I (indicated in Supplementary Figure S1) were obtained. Arrows indicate the position of each primer used in primer-extension experiments. When a CG site is methylated or unmethylated, either “C” or “T” is appended, respectively. (B) Representative image of the Ms-SNuPE analysis at the CG-II site. Purified bisulfite-PCR products (25 ng), a primer, and radioisotope-labeled C or T were reacted for primer extension; products were purified and loaded on polyacrylamide gels for electrophoresis (PAGE). A methylation value for each CG site was computed using the following equation: methylated C/(methylated C + unmethylated T)×100. GN sample numbers, T830, T1052, and T334, reflect GN1, GN2, and GN3 in Figure 1D.
(0.46 MB TIF)

Figure S4 Representative images from immunohistochemical staining for LAPTM5, H-Ras, or CD20 (BCA-B20) in degenerating NB cells within regressing areas in mass-screened NB tumors (A) Representative images of immunostaining for each protein in regressing areas within sections from Stage-4S NB tumors detected by mass-screening. Serial tumor sections were stained with hematoxylin-eosin (HE) (left), with LAPTM5 (middle), or with H-Ras antibody (right). Arrowheads indicate a degenerated area. (B) Representative images of CD20 (BCA-B20) staining in regressing areas within sections from Stage-1 NB tumors detected by mass-screening. Arrowheads indicate CD20-positive hematopoietic cells.
(5.55 MB TIF)

Figure S5 Detection of cleaved caspase-3 during CDDP- or LAPTM5-induced cell death (A) Representative image of cleaved caspase-3 staining. The cells (2×105/well for CDDP, 1×105/well for infection) were plated on coverslips in 24-well plates, and the next day treated with Cisplatin (CDDP; 1.5 µg/ml in SH-SY5Y cells) for 2 days or infected with Ad-LacZ or Ad-LAPTM5 at a MOI of 10 for 4 days. Cells were fixed, reacted with an antibody to cleaved caspase-3, and visualized with a Alexa Fluor 594-conjugated secondary antibody. (B) Quantitation of the percentage of cleaved caspase-3-positive cells during cell death in NB cell lines including SH-SY5Y in (A). The percentage of cleaved caspase-3-positive cells among all cells (at least 500 nuclei) was counted. Vertical lines, SD for three experiments. (C) Western blotting for cleaved caspase-3. The cells were treated as indicated in (A) and whole-cell lysate was analyzed by immunoblotting with an antibody to cleaved caspase-3 or β-actin (internal control). (D) Effect of zVAD-fmk treatment during CDDP-induced cell death in SH-SY5Y cells. SH-SY5Y (1×104/well) cells plated in 96-well plates were treated with CDDP (1.5 ug/ml) in the absence or presence of zVAD-fmk (100 µM). The percentage of surviving cells was determined two days after treatment by WST assay. Vertical lines, SD for three experiments. *t-test. p<0.05
(1.16 MB TIF)

Figure S6 Appearance of autophagic vacuoles during LAPTM5-induced cell death in SH-SY5Y and GOTO cells stably expressing GFP-LC3 (A) Localization of endogenous LC3B detected by immunofluorescence microscopy. SH-SY5Y cells (1×105/well) were plated on coverslips in 24-well plates, and the next day infected with Ad-LacZ or Ad-LAPTM5 at a MOI of 10. Four days later, the cells were fixed in 10% TCA 4 days after infection, reacted with the LC3B antibody, and visualized with an Alexa Fluor 594-conjugated secondary antibody. (B) Detection of LC3 form-II by western blotting. As the time-courses indicated, whole-cell lysate from SH-SY5Y cells (left) or GOTO cells stably expressing GFP-LC3 (right) infected with Ad-LacZ and Ad-LAPTM5 at a MOI of 10 was analyzed by immunoblotting with an antibody to endogenous LC3B, GFP, or β-actin (internal control). Arrows indicate form-I or -II of LC3B or GFP-LC3.
(0.98 MB TIF)

Figure S7 Representative images of the subcellular localization of GFP-LC3 or endogenous LC3B by immunofluorescence microscopy GOTO cells stably expressing GFP-LC3 (A) or parental GOTO cells (B) were prepared and treated as described in Figure 4B. The number of cells with a GFP-LC3 or LC3B punctate pattern was decreased by treatment with wortmannin (Wort.; 200 nM) or knockdown of ATG5 during starvation or LAPTM5-induced cell death.
(2.30 MB TIF)

Figure S8 Accumulation of p62/SQSTM1 protein in Ad-LAPTM5-infected GOTO cells. The accumulation of p62/SQSTM1 protein in Ad-LAPTM5-infected GOTO cells was revealed by immunofluorescence microscopy. Four days after infection, cells were fixed, reacted with an antibody to p62/SQSTM1, and visualized with Alexa Fluor 594-conjugated secondary antibody. The images were obtained with different exposure times (0.2 and 0.02 seconds for Ad-LacZ and Ad-LAPTM5-infected cells, respectively).
(0.64 MB TIF)

Figure S9 Subcellular localization of accumulated LAPTM5 during LAPTM5-induced cell death. Representative image of the co-staining of LAMP2 (Green) and LAPTM5 (Red). GOTO cells were infected with Ad-LacZ or Ad-LAPTM5 at a MOI of 10 in medium containing Bafilomycin A1 (Baf.A1, 2 nM) or ALLN (2 µM) for 2 days. Immunofluorescence microscopy was performed as in Figure 6A. DAPI was counterstained. In GOTO cells subjected to mild treatment with Baf.A1 or ALN, LAPTM5 was frequently localized into LAMP2-positive lysosomes.
(4.81 MB TIF)

Figure S10 Lysosomal membrane permeabilization (LMP) during LAPTM5-induced cell death. (A) Frequency of cells with loss of a punctuate pattern of LTR staining. One or four days after infection with Ad-LacZ or Ad-LAPTM5, cells that had lost the punctuate pattern of LTR staining, so-called “Pale cells”, were counted, and the number recorded as a percentage of all cells. The graph indicates the percentages of cells in both cell lines. Vertical lines, SD for three separate experiments. *t-test, p<0.001, **p<0.05. No inf., no infection. (B) Representative images of staining for FITC-dextran (40 kD) in infected GOTO cells. GOTO cells (1×105/well) were plated on coverslips in 24-well plates, and the next day infected with Ad-LacZ or Ad-LAPTM5 at a MOI of 10, and then maintained in medium containing 100 µg/ml of FITC-dextran (40kD). Right; Four days after infection, cells were washed twice, fixed in 4% formaldehyde, and observed by fluorescence microscope. Left; The percentage of cells that released FITC-dextran (40 kD) among cells at least 200 cells was measured. Vertical lines, SD for three separate experiments. *t-test. p<0.05.
(1.10 MB TIF)

Figure S11 Detection of GFP-LC3 form-II and accumulation of p62/SQSTM1 on treatment with Ciprofloxacin (CPX) GOTO cells stably expressing GFP-LC3 (1×106/well) were plated in 6-well plates, and treated with CPX (100 or 300 µg/ml) for one day. As a solvent for CPX, 0.1N HCl was added with 1 or 3% of the medium. Whole-cell lysate from treated cells was analyzed by immunoblotting with antibodies to the indicated proteins.
(0.43 MB TIF)

Figure S12 Detection of endogenous LAPTM5 by western blotting. (A) Relative levels of LAPTM5 mRNA levels during induction of differentiation in IMR32 cells. Cells were induced to neuronal differentiation by maintaining in serum-free medium including retinoic acid (RA,10 µM) for 4 days. The levels of LAPTM5 mRNA at the indicated days were determined by quantitative RT-PCR and normalized with each level of GAPDH mRNA. Vertical lines, SD. (B) Morphological change during RA-induced differentiation and treatment with lysosomal or proteasomal inhibitor. IMR32 cells (2×106/well) were plated in 10 cm dishes and maintained in serum-free medium including RA (10 µM) (SFRA). At 3 days after induction, Baf.A1 (25 nM) or ALLN (10 µM) were added in medium and then cultured for 1 day. Treatment with SFRA efficiently induced the appearance of cells with a number of axonal outgrowth in IMR32 cells (a; parental IMR32 cells and b; differentiated IMR32 cells). Additional treatment with Baf.A1 (c) or ALLN (d) frequently induced cell death with a loss of axon. (C) Detection of endogenous LAPTM5 protein by western blotting. Cells were treated as indicated in (b). As a positive control for detection, whole-cell lysate from LCL highly expressing with or without Baf.A1 (10 nM, 1 day) or ALLN (10 µM 1 day) was used. Arrows indicate bands for endogenous LAPTM5 detected in expected size. TBS buffer including 0.05% Tween-20 and 1% Casein was used for blocking and reaction with antibody. Endogenous LAPTM5 was detected in expected size. Astarisks indicate non-specific bands. The results shown represent two independent experiments.
(2.52 MB TIF)
