# Supplementary material for: Lysosomal-Associated Protein Multispanning Transmembrane 5 Gene (LAPTM5) Is Associated with Spontaneous Regression of Neuroblastomas
Source: PLoS One. 2009 Sep 29;4(9):e7099. doi: 10.1371/journal.pone.0007099 (PMC2746316; doi:10.1371/journal.pone.0007099)
Supplement: Methods S1 — Supplementary methods and Supplementary references (0.03 MB DOC) [file pone.0007099.s001.doc]

**Supplementary Methods**

BAC array-based MCA (BAMCA)

BAMCA using our in-house 1p35-p36 contig-array, which contains 208 BAC clones covering almost the entire 1p35-p36 region, was carried out as described elsewhere (1,2). The hybridized arrays were scanned with a GenePix 4000B fluorescence scanner (Axon Instruments), and analyzed using GenePix Pro 4.1 software (Axon Instruments). Positive spots containing differentially methylated sequences were detected as a ratio of Cy3 (test) to y5 (reference) of greater than 1.0 after deduction of the values of BAC spots without *Sma*I sites as background, and global normalization.

**Methylation analysis by methylation sensitive (MS)-PCR and combined bisulfite restriction analysis (COBRA)**

Genomic DNA (1 µg) was digested with a methylation-sensitive *Sma*I (NEB) at 25˚C for 24 h, and then amplified using appropriate primers. Methylation status at each *Sma*I site was determined by the presence or absence of a PCR product. For COBRA, genomic DNA was treated with sodium bisulfite and amplified with primers for sequences of interest. PCR products were digested with each restriction enzyme. Primer sequences used for the methylation analysis are provided in Supplementary Table S3.

**Fluorescence in situ hybridization (FISH) analysis**

Metaphase chromosomes were prepared from NB cell lines by standard methods. FISH analyses were performed as described previously (3).

**Array-CGH analysis**

The MCG Cancer Array-800 (1), which contains 800 BAC/PAC clones that include known cancer-related genes, was used for the analysis of copy-numbers in NB cell lines. Hybridizations was carried out as described elsewhere (3), and arrays were scanned with a GenePix 4000B (Axon Instruments). Acquired images were analyzed with GenePix Pro 6.1 imaging software (Axon Instruments). Fluorescence ratios were normalized so that the mean of the middle third of log_2_ ratios across the array was zero. Average ratios that deviated significantly from zero (>2 SD) were considered abnormal.

**Fluorescein isothiocyanate-labelled dextran (FITC-dextran; 40kD) staining**Cells were maintained in medium containing 100 μg/ml of FITC-dextran (40kD) during adenovirus infection. Four days after infection, cells were washed twice, fixed in 4% formaldehyde, and observed by fluorescence microscope. The percentage of cells that released FITC-dextran (40 kD) into the cytosol among cells at least 200 cells was measured.

Western blotting for detection endogenous LAPTM5 protein

Whole-cell lysate was prepared in lysis buffer (20 mM Hepes pH 7.4, 120 mM NaCl, 5 mM EDTA, 10% glycerol, and 1% Triton-X 100) containing a proteinase inhibitor cocktail (Roche). Whole-cell lysate (80 μg) was analyzed by immunoblotting. TBS buffer including 0.05% Tween-20 and 1% Casein was used as the solution for blocking and dilution of antibodies. Whole-cell lysate (10 μg) from LCL highly expressing LAPTM5 was used as a positive control for western blotting.

Reagents

Ciprofloxican (CPX) was obtained from LKT laboratories. Retinoic acid (RA) and FITC-dextran (40kD) were from Sigma.

**Supplementary references**

1. Inazawa J, Inoue J, Imoto I (2004) Comparative genomic hybridization (CGH)-arrays pave the way for identification of novel cancer-related genes. Cancer Sci 95: 559-563.
2. Misawa A, Inoue J, Sugino Y, Hosoi H, Sugimoto T, et al. (2005) Methylation-associated silencing of the nuclear receptor 1I2 gene in advanced-type neuroblastomas, identified by bacterial artificial chromosome array-based methylated CpG island amplification. Cancer Res 65: 10233-10242.
3. Sonoda I, Imoto I, Inoue J, Shibata T, Shimada Y, et al. (2004) Frequent silencing of low density lipoprotein receptor-related protein 1B (LRP1B) expression by genetic and epigenetic mechanisms in esophageal squamous cell carcinoma. Cancer Res 64: 3741-3747.
