# Supplementary figures and images for: Lysosomal-Associated Protein Multispanning Transmembrane 5 Gene (LAPTM5) Is Associated with Spontaneous Regression of Neuroblastomas
Source: PLoS One. 2009 Sep 29;4(9):e7099. doi: 10.1371/journal.pone.0007099 (PMC2746316; doi:10.1371/journal.pone.0007099)

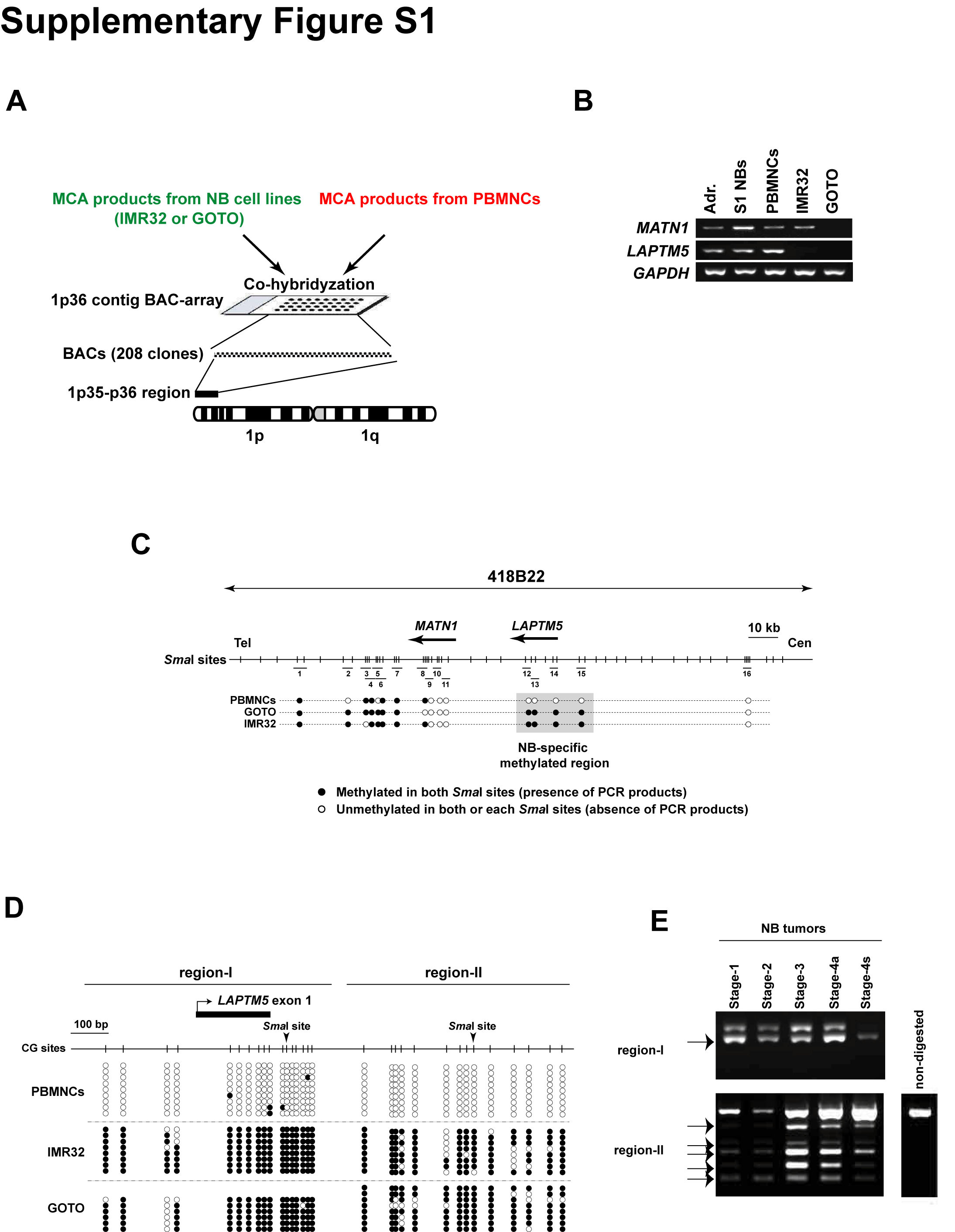

Supplement: Figure S1 — Validation of candidate genes down-regulated through DNA methylation (A) Schematic diagram of BAMCA experiments. DNA fragments generated by the MCA method from NB cell lines (Green) and from the references (Red) were labeled with Cy3 or Cy5 respectively, and co-hybridized on the in-house1p35–p36 contig array. (B) Screening of down-regulated genes by RT-PCR analysis. GAPDH served as an internal control. Adr, normal adrenal glands; S1 NBs, pooled samples from five stage-1 NB tumors. (C) Determination of methylation status of SmaI sites within 418B22, a BAC clone that includes LAPTM5, by MS-PCR in PBMNCs and the two NB cell lines GOTO and IMR32. Sixteen primer sets were used for PCR in genomic regions including at least two SmaI sites in a distance range of 200–2,000 bp, because the MCA procedure produced a genomic fragment having two adjacent methylated SmaI sites at each end. Black and white circles indicate the presence (methylated) or absence (unmethylated) of PCR products, respectively. The region (including no. 12–16) shown by a gray box indicates the NB-specific methylated region at the LAPTM5 locus. (D) Determination of methylation status at CG sites around the transcriptional-start site of the LAPTM5 gene by bisulfite sequencing. A total of 32 CG sites within 1 kb of the start site were analyzed using two primer sets (regions-I and -II). An arrow at the start-site shows the direction of transcription. Arrowheads indicate two SmaI sites that located within the PCR product produced by no. 14 primers, as indicated in (C). GOTO and IMR32 cells were both widely methylated, whereas PBMNCs and an EBV-transformed lymphocyte cell line (LCL) were almost completely unmethylated. (E) Representative images of COBRA for LAPTM5 in primary NB tumors. Methylation of this gene was detected in regions-I and -II in all NB tumors examined. Bisulfite-PCR products for each of the two regions indicated in (D) were digested with HinfI (region-I) and TaqI (region-II). Arrows indicate w [file pone.0007099.s005.tif]

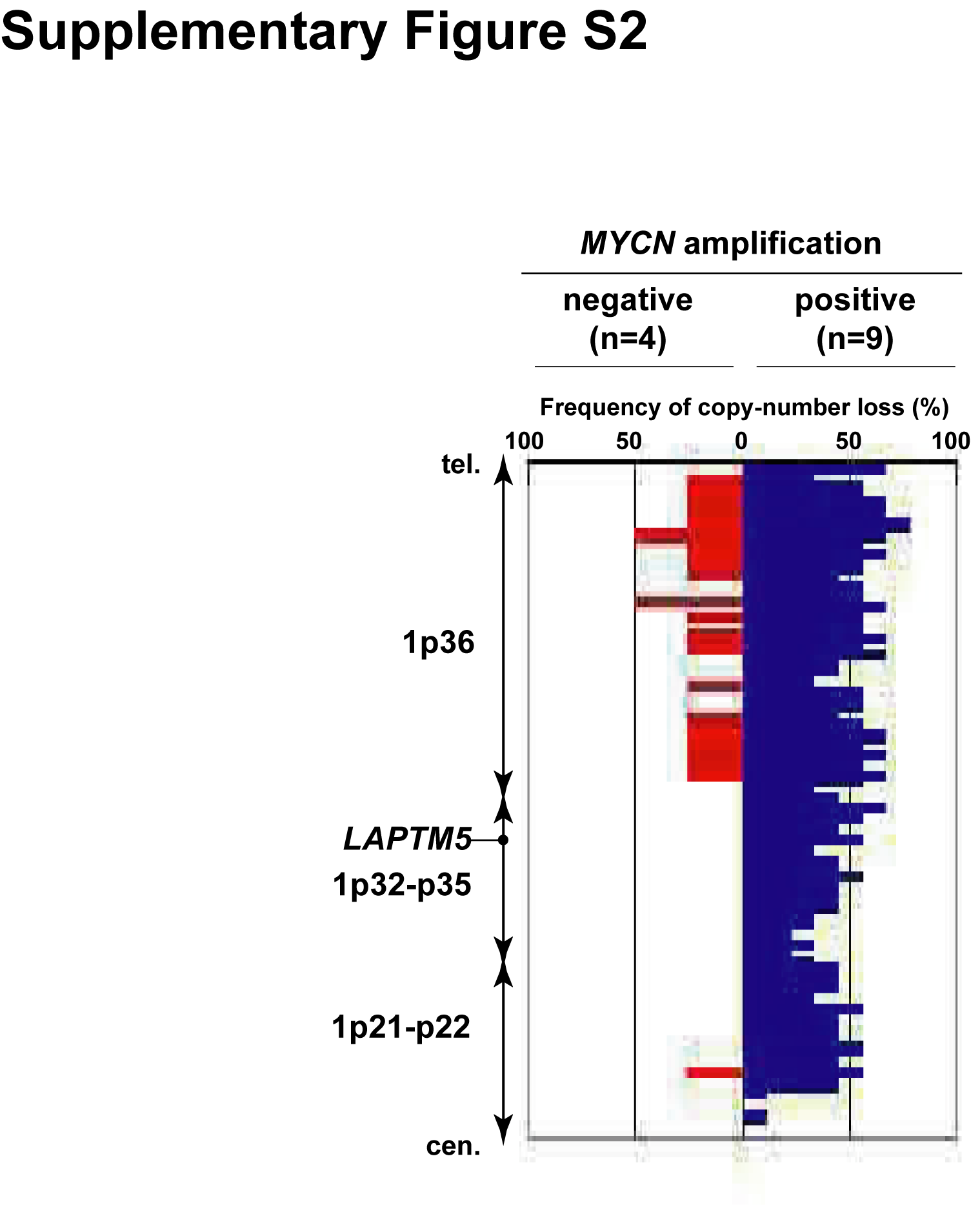

Supplement: Figure S2 — Analysis of DNA copy-number aberrations in the 1p region in NB cell lines Frequency of 1p loss in NB cell lines revealed by array-CGH. In house ‘MCG cancer array-800’-spotted arbitrary BACs, including 43 BACs on 1p, were used for analyzing 13 NB cell lines (GOTO, IMR32, SJ-N-CG, CHP134, MP-N-MS, KP-N-RT, SMS-KCN, SK-N-DZ, SMS-KAN, SK-N-AS, SH-SY5Y, SK-N-SH, and SK-N-KP). The sideways red (4 non-amplified NB cell lines) or blue (MYCN-amplified NB cell lines) bars indicate frequencies of loss on each BAC, respectively. A large range of loss on 1p, including the LAPTM5 locus, was detected frequently in MYCN-amplified NB cell lines. (0.93 MB TIF) [file pone.0007099.s006.tif]

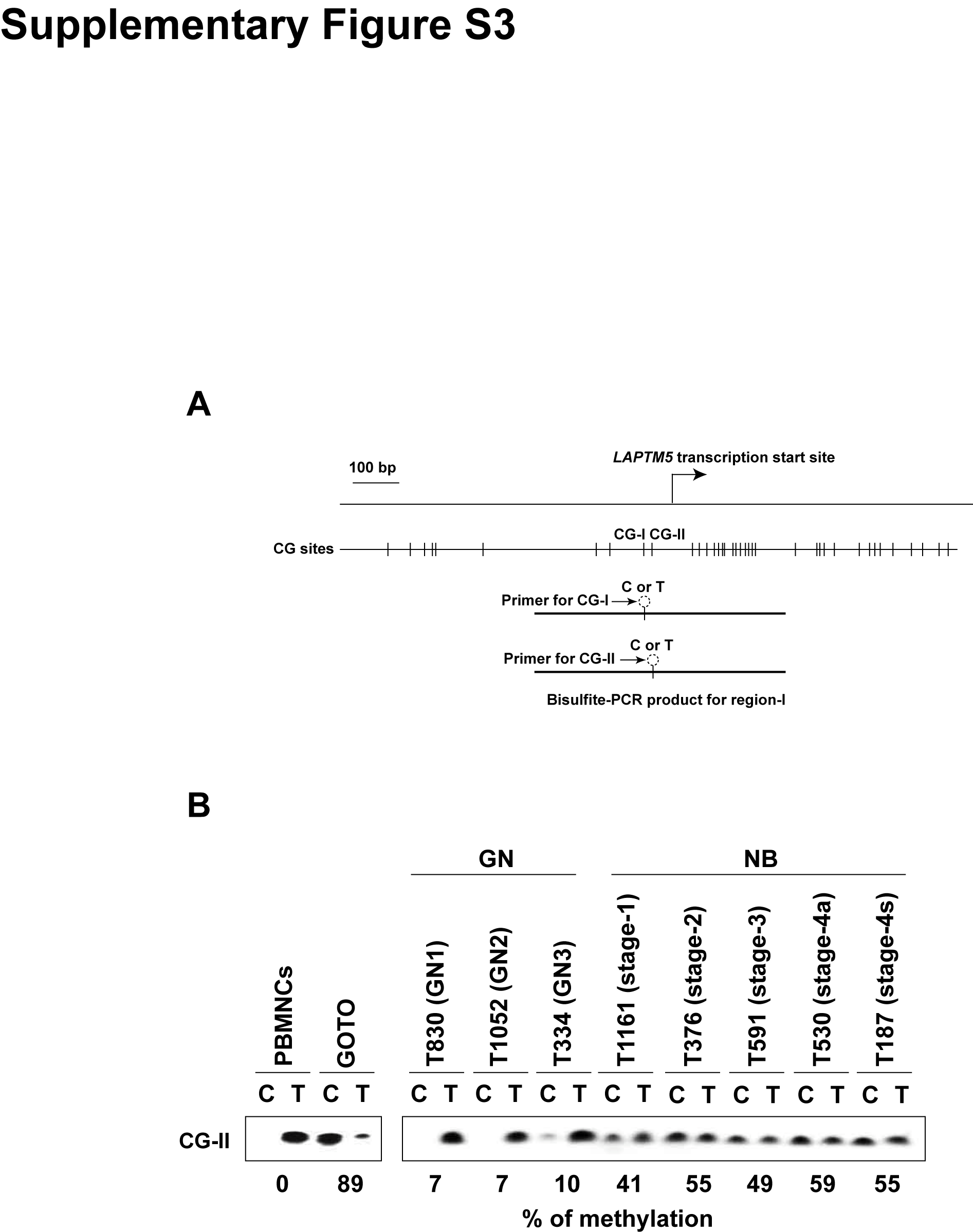

Supplement: Figure S3 — Methylation analysis in primary NB tumors and ganglioneuromas (GNs) by the methylation-sensitive single-nucleotide primer extension (Ms-SNuPE) method (A) Positions of primers used in the Ms-SNuPE analysis. Bisulfite-PCR products for region-I (indicated in Supplementary Figure S1) were obtained. Arrows indicate the position of each primer used in primer-extension experiments. When a CG site is methylated or unmethylated, either “C” or “T” is appended, respectively. (B) Representative image of the Ms-SNuPE analysis at the CG-II site. Purified bisulfite-PCR products (25 ng), a primer, and radioisotope-labeled C or T were reacted for primer extension; products were purified and loaded on polyacrylamide gels for electrophoresis (PAGE). A methylation value for each CG site was computed using the following equation: methylated C/(methylated C + unmethylated T)×100. GN sample numbers, T830, T1052, and T334, reflect GN1, GN2, and GN3 in Figure 1D. (0.46 MB TIF) [file pone.0007099.s007.tif]

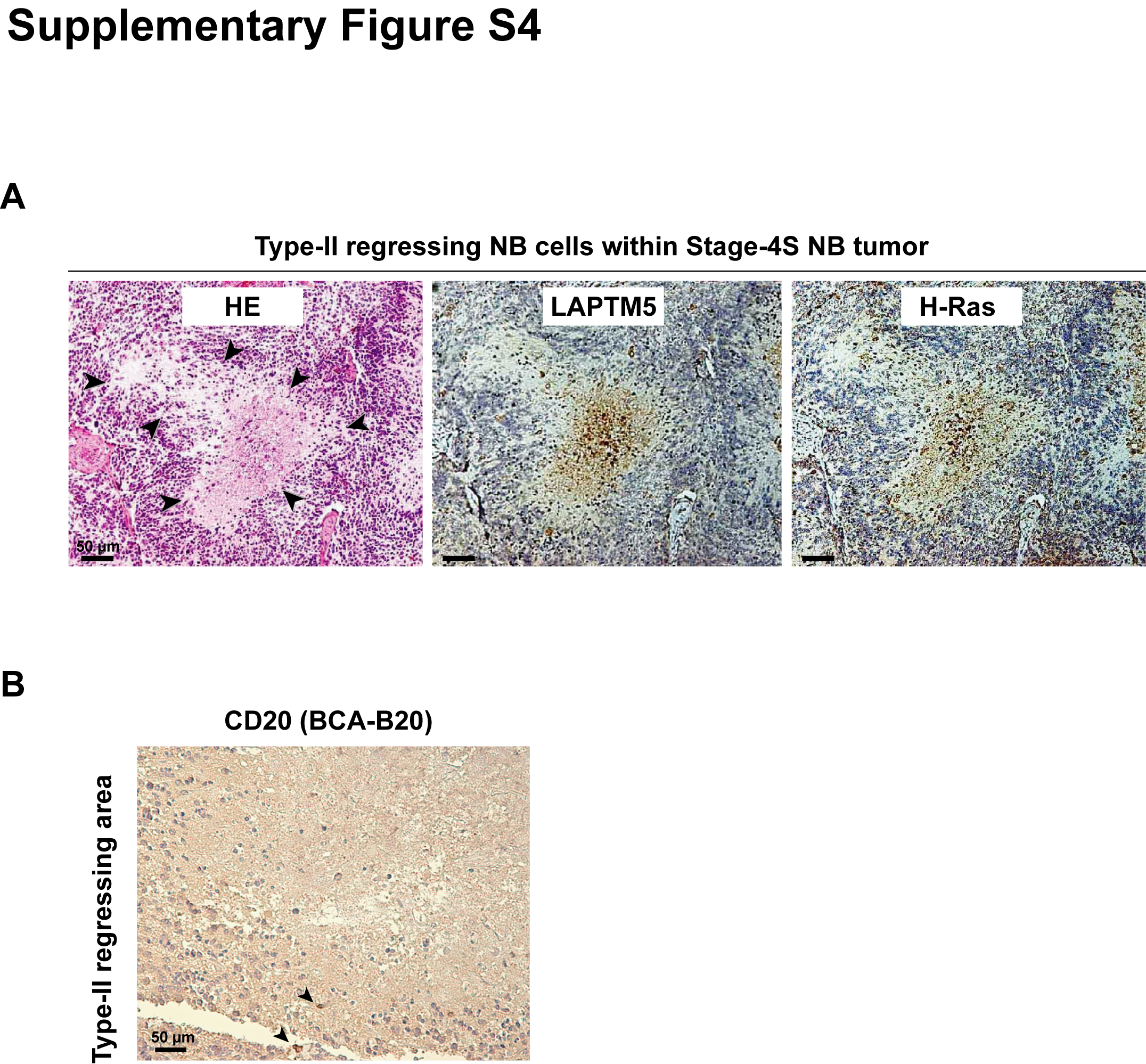

Supplement: Figure S4 — Representative images from immunohistochemical staining for LAPTM5, H-Ras, or CD20 (BCA-B20) in degenerating NB cells within regressing areas in mass-screened NB tumors (A) Representative images of immunostaining for each protein in regressing areas within sections from Stage-4S NB tumors detected by mass-screening. Serial tumor sections were stained with hematoxylin-eosin (HE) (left), with LAPTM5 (middle), or with H-Ras antibody (right). Arrowheads indicate a degenerated area. (B) Representative images of CD20 (BCA-B20) staining in regressing areas within sections from Stage-1 NB tumors detected by mass-screening. Arrowheads indicate CD20-positive hematopoietic cells. (5.55 MB TIF) [file pone.0007099.s008.tif]

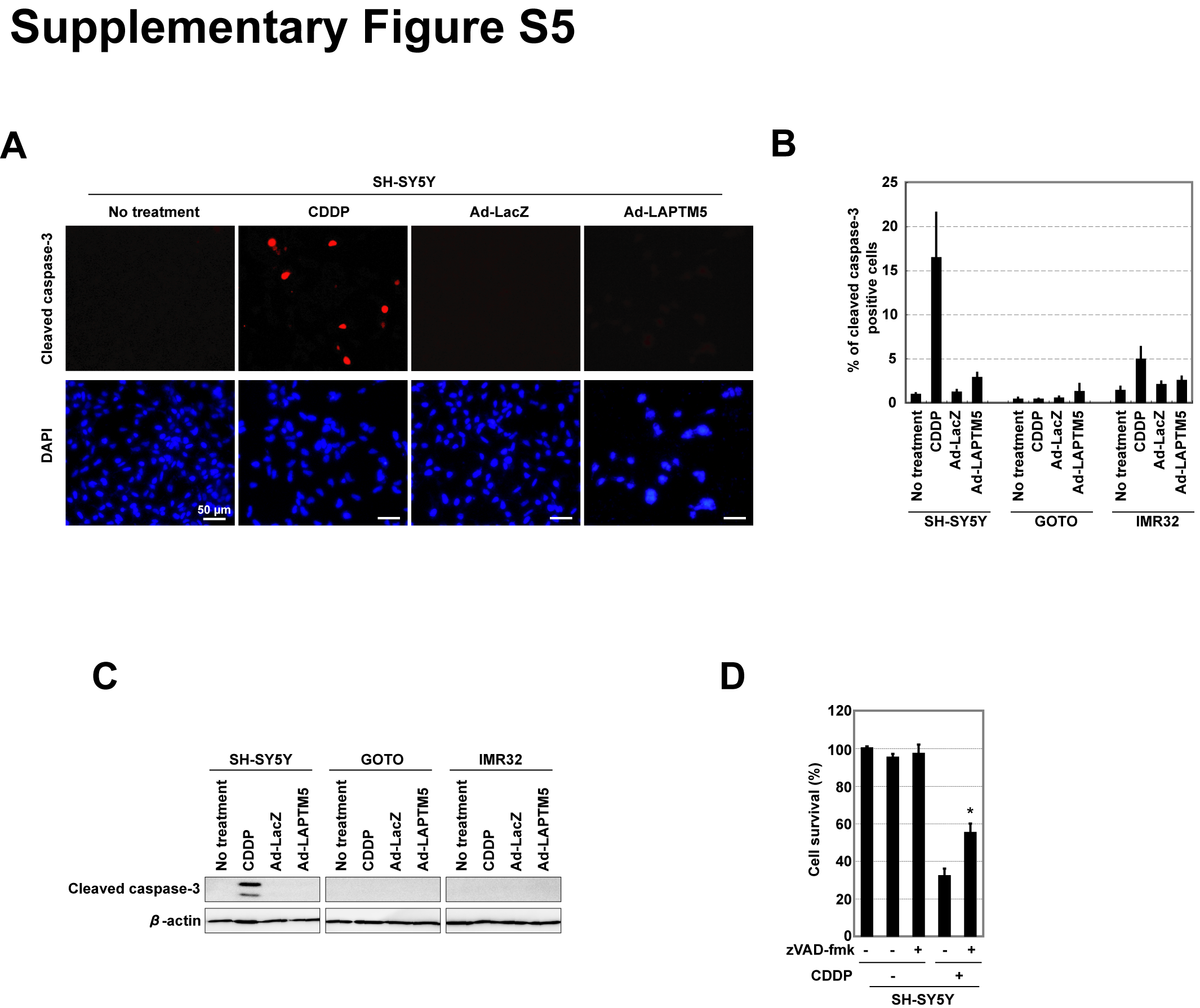

Supplement: Figure S5 — Detection of cleaved caspase-3 during CDDP- or LAPTM5-induced cell death (A) Representative image of cleaved caspase-3 staining. The cells (2×10^5^/well for CDDP, 1×10^5^/well for infection) were plated on coverslips in 24-well plates, and the next day treated with Cisplatin (CDDP; 1.5 µg/ml in SH-SY5Y cells) for 2 days or infected with Ad-LacZ or Ad-LAPTM5 at a MOI of 10 for 4 days. Cells were fixed, reacted with an antibody to cleaved caspase-3, and visualized with a Alexa Fluor 594-conjugated secondary antibody. (B) Quantitation of the percentage of cleaved caspase-3-positive cells during cell death in NB cell lines including SH-SY5Y in (A). The percentage of cleaved caspase-3-positive cells among all cells (at least 500 nuclei) was counted. Vertical lines, SD for three experiments. (C) Western blotting for cleaved caspase-3. The cells were treated as indicated in (A) and whole-cell lysate was analyzed by immunoblotting with an antibody to cleaved caspase-3 or β-actin (internal control). (D) Effect of zVAD-fmk treatment during CDDP-induced cell death in SH-SY5Y cells. SH-SY5Y (1×10^4^/well) cells plated in 96-well plates were treated with CDDP (1.5 ug/ml) in the absence or presence of zVAD-fmk (100 µM). The percentage of surviving cells was determined two days after treatment by WST assay. Vertical lines, SD for three experiments. *t-test. p<0.05 (1.16 MB TIF) [file pone.0007099.s009.tif]

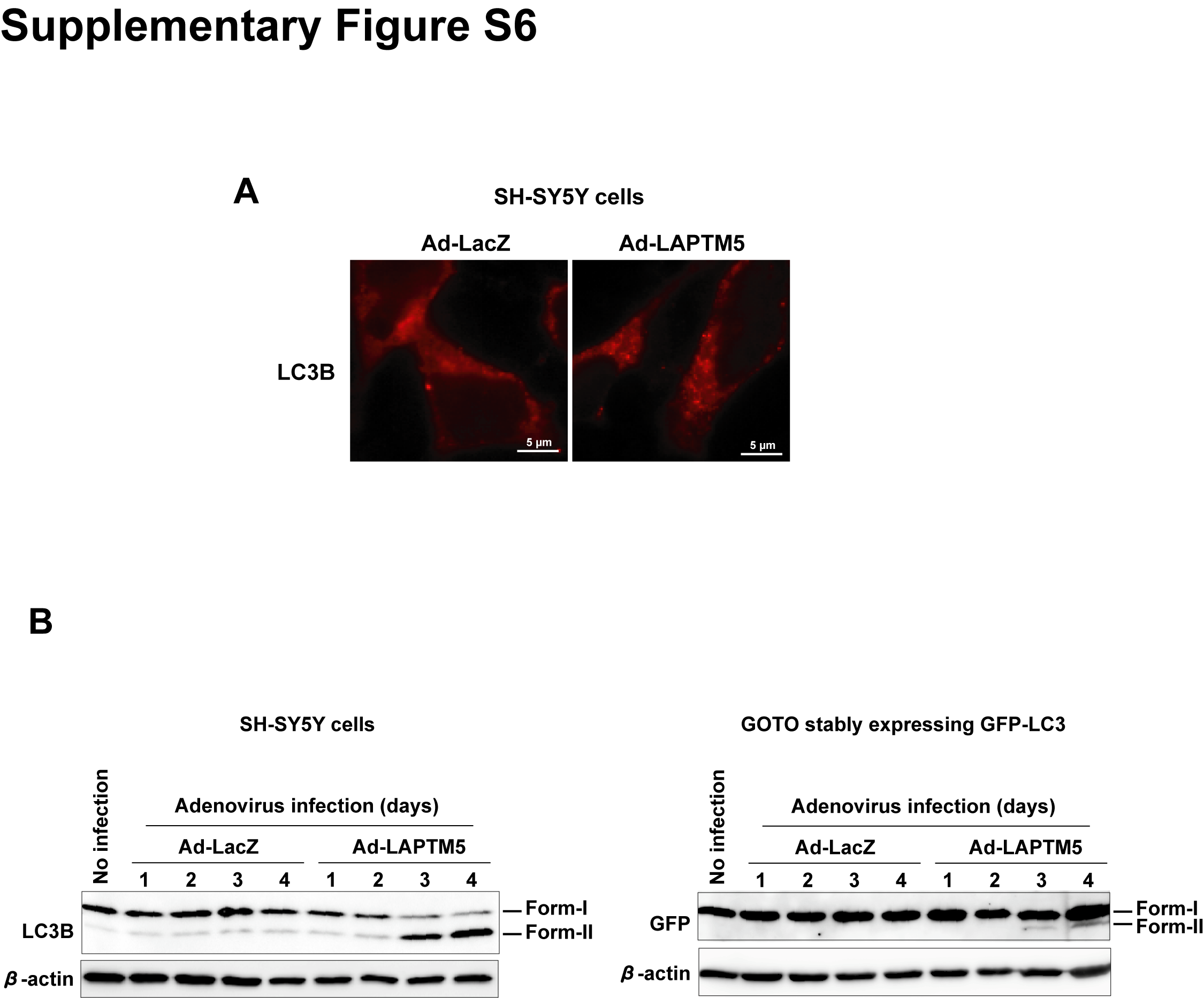

Supplement: Figure S6 — Appearance of autophagic vacuoles during LAPTM5-induced cell death in SH-SY5Y and GOTO cells stably expressing GFP-LC3 (A) Localization of endogenous LC3B detected by immunofluorescence microscopy. SH-SY5Y cells (1×10^5^/well) were plated on coverslips in 24-well plates, and the next day infected with Ad-LacZ or Ad-LAPTM5 at a MOI of 10. Four days later, the cells were fixed in 10% TCA 4 days after infection, reacted with the LC3B antibody, and visualized with an Alexa Fluor 594-conjugated secondary antibody. (B) Detection of LC3 form-II by western blotting. As the time-courses indicated, whole-cell lysate from SH-SY5Y cells (left) or GOTO cells stably expressing GFP-LC3 (right) infected with Ad-LacZ and Ad-LAPTM5 at a MOI of 10 was analyzed by immunoblotting with an antibody to endogenous LC3B, GFP, or β-actin (internal control). Arrows indicate form-I or -II of LC3B or GFP-LC3. (0.98 MB TIF) [file pone.0007099.s010.tif]

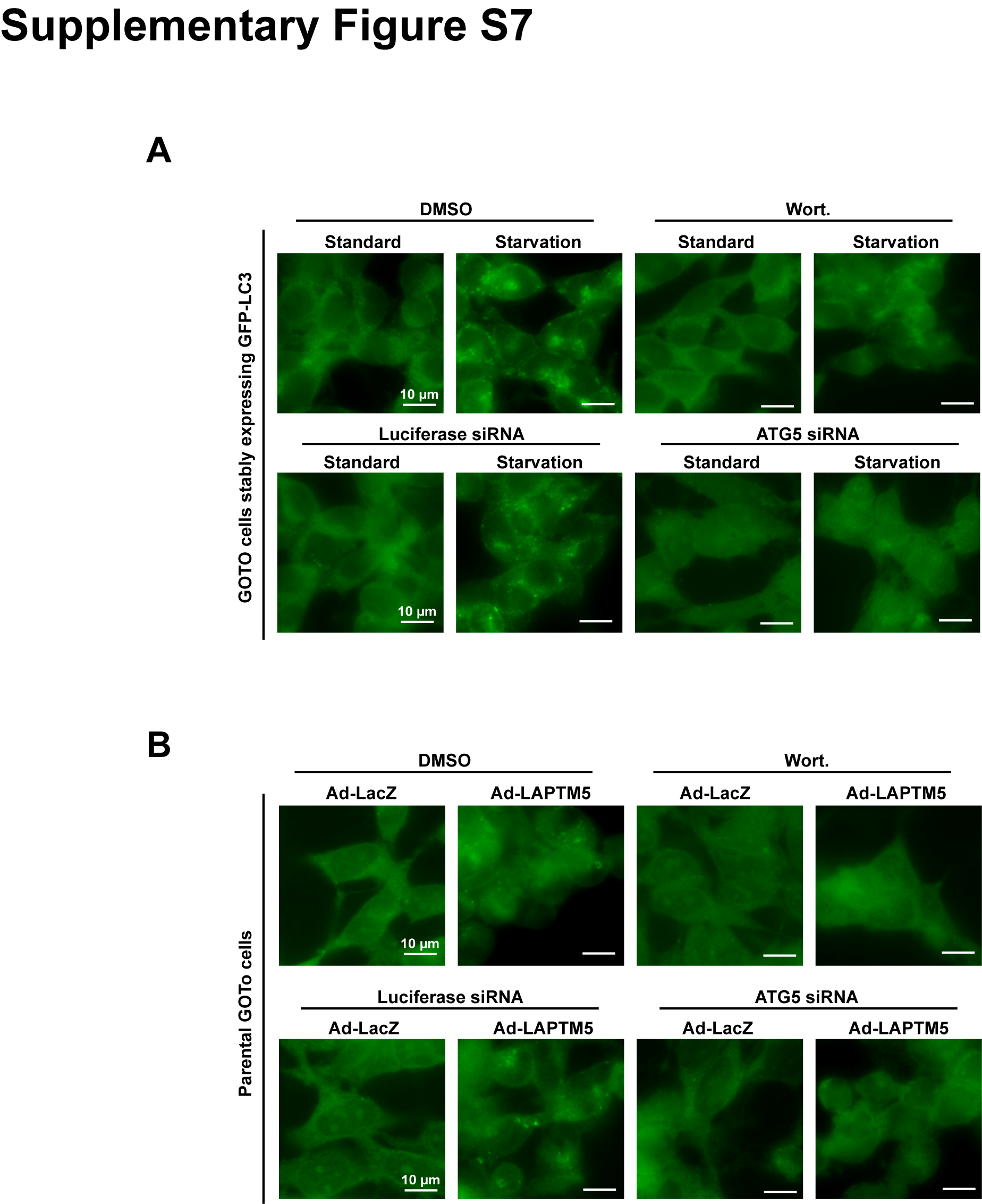

Supplement: Figure S7 — Representative images of the subcellular localization of GFP-LC3 or endogenous LC3B by immunofluorescence microscopy GOTO cells stably expressing GFP-LC3 (A) or parental GOTO cells (B) were prepared and treated as described in Figure 4B. The number of cells with a GFP-LC3 or LC3B punctate pattern was decreased by treatment with wortmannin (Wort.; 200 nM) or knockdown of ATG5 during starvation or LAPTM5-induced cell death. (2.30 MB TIF) [file pone.0007099.s011.tif]

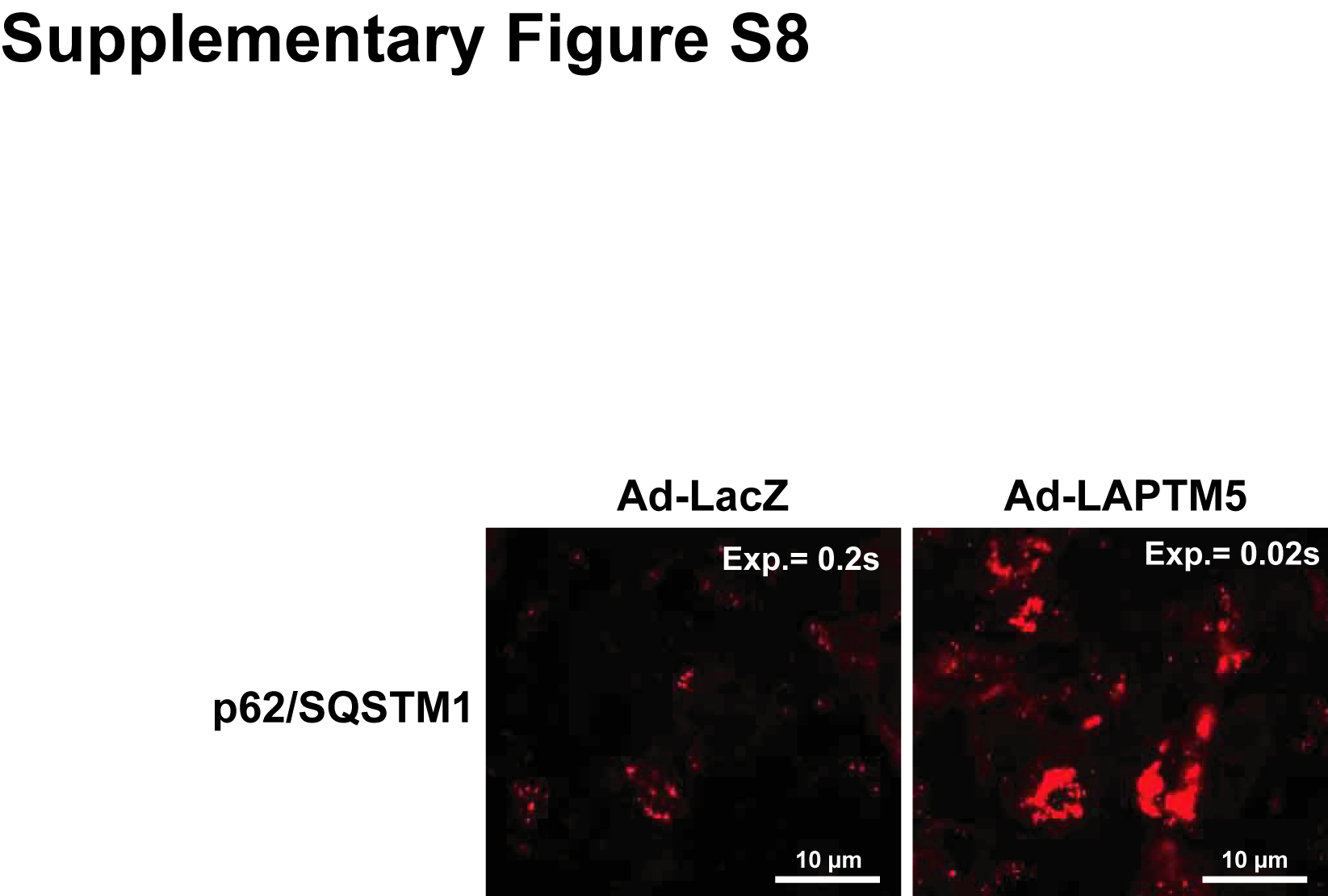

Supplement: Figure S8 — Accumulation of p62/SQSTM1 protein in Ad-LAPTM5-infected GOTO cells. The accumulation of p62/SQSTM1 protein in Ad-LAPTM5-infected GOTO cells was revealed by immunofluorescence microscopy. Four days after infection, cells were fixed, reacted with an antibody to p62/SQSTM1, and visualized with Alexa Fluor 594-conjugated secondary antibody. The images were obtained with different exposure times (0.2 and 0.02 seconds for Ad-LacZ and Ad-LAPTM5-infected cells, respectively). (0.64 MB TIF) [file pone.0007099.s012.tif]

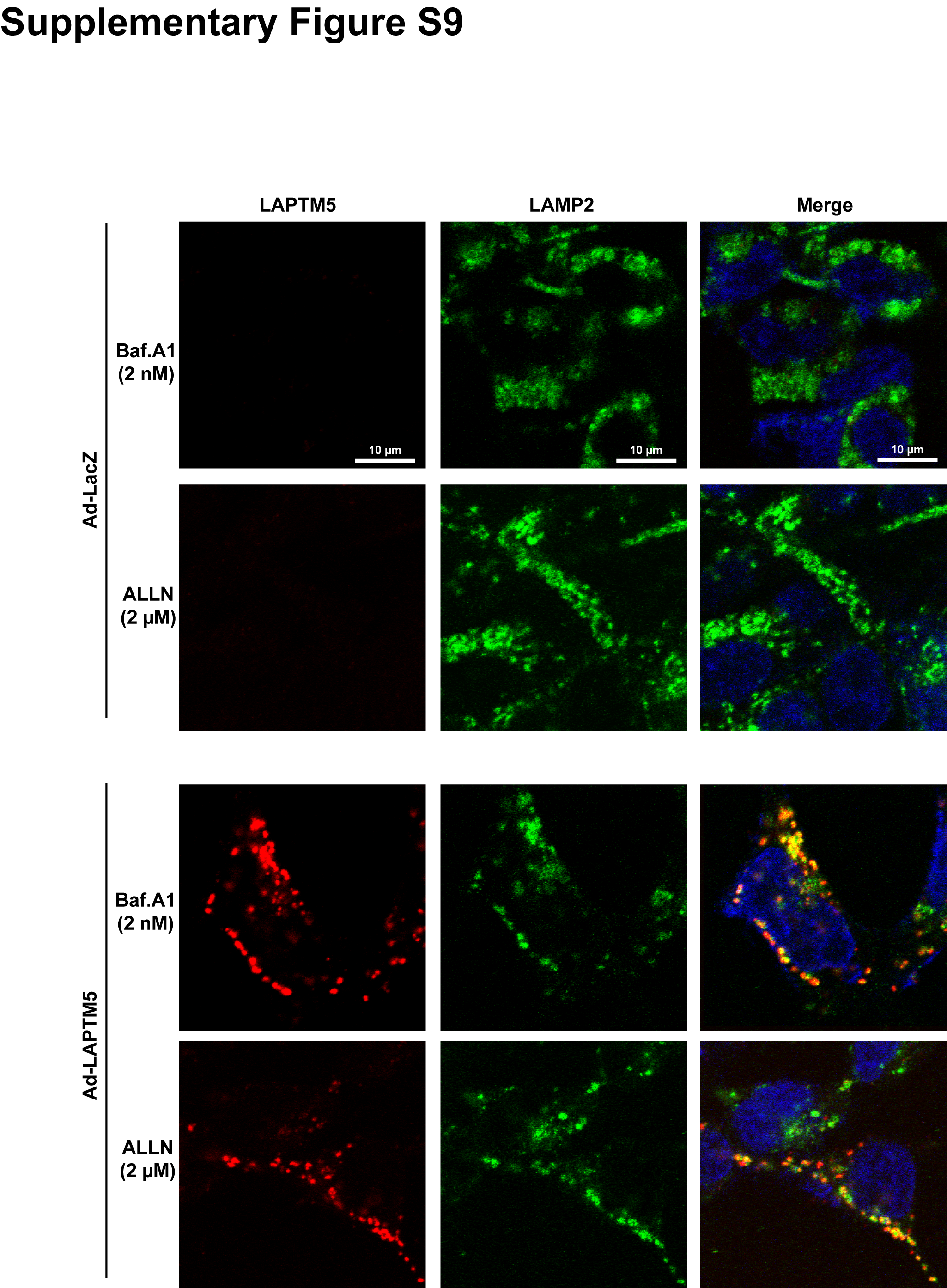

Supplement: Figure S9 — Subcellular localization of accumulated LAPTM5 during LAPTM5-induced cell death. Representative image of the co-staining of LAMP2 (Green) and LAPTM5 (Red). GOTO cells were infected with Ad-LacZ or Ad-LAPTM5 at a MOI of 10 in medium containing Bafilomycin A1 (Baf.A1, 2 nM) or ALLN (2 µM) for 2 days. Immunofluorescence microscopy was performed as in Figure 6A. DAPI was counterstained. In GOTO cells subjected to mild treatment with Baf.A1 or ALN, LAPTM5 was frequently localized into LAMP2-positive lysosomes. (4.81 MB TIF) [file pone.0007099.s013.tif]

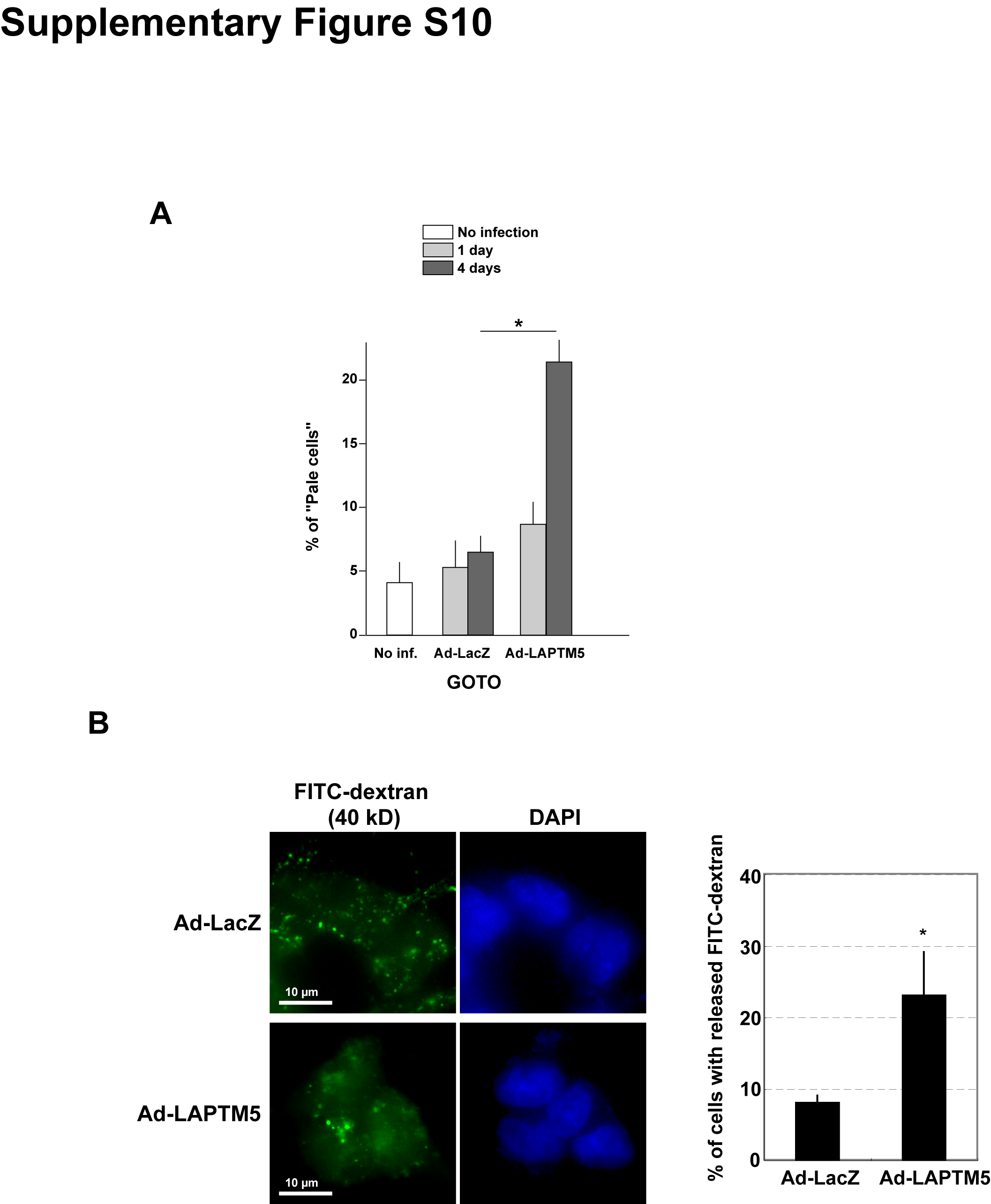

Supplement: Figure S10 — Lysosomal membrane permeabilization (LMP) during LAPTM5-induced cell death. (A) Frequency of cells with loss of a punctuate pattern of LTR staining. One or four days after infection with Ad-LacZ or Ad-LAPTM5, cells that had lost the punctuate pattern of LTR staining, so-called “Pale cells”, were counted, and the number recorded as a percentage of all cells. The graph indicates the percentages of cells in both cell lines. Vertical lines, SD for three separate experiments. *t-test, p<0.001, **p<0.05. No inf., no infection. (B) Representative images of staining for FITC-dextran (40 kD) in infected GOTO cells. GOTO cells (1×10^5^/well) were plated on coverslips in 24-well plates, and the next day infected with Ad-LacZ or Ad-LAPTM5 at a MOI of 10, and then maintained in medium containing 100 µg/ml of FITC-dextran (40kD). Right; Four days after infection, cells were washed twice, fixed in 4% formaldehyde, and observed by fluorescence microscope. Left; The percentage of cells that released FITC-dextran (40 kD) among cells at least 200 cells was measured. Vertical lines, SD for three separate experiments. *t-test. p<0.05. (1.10 MB TIF) [file pone.0007099.s014.tif]

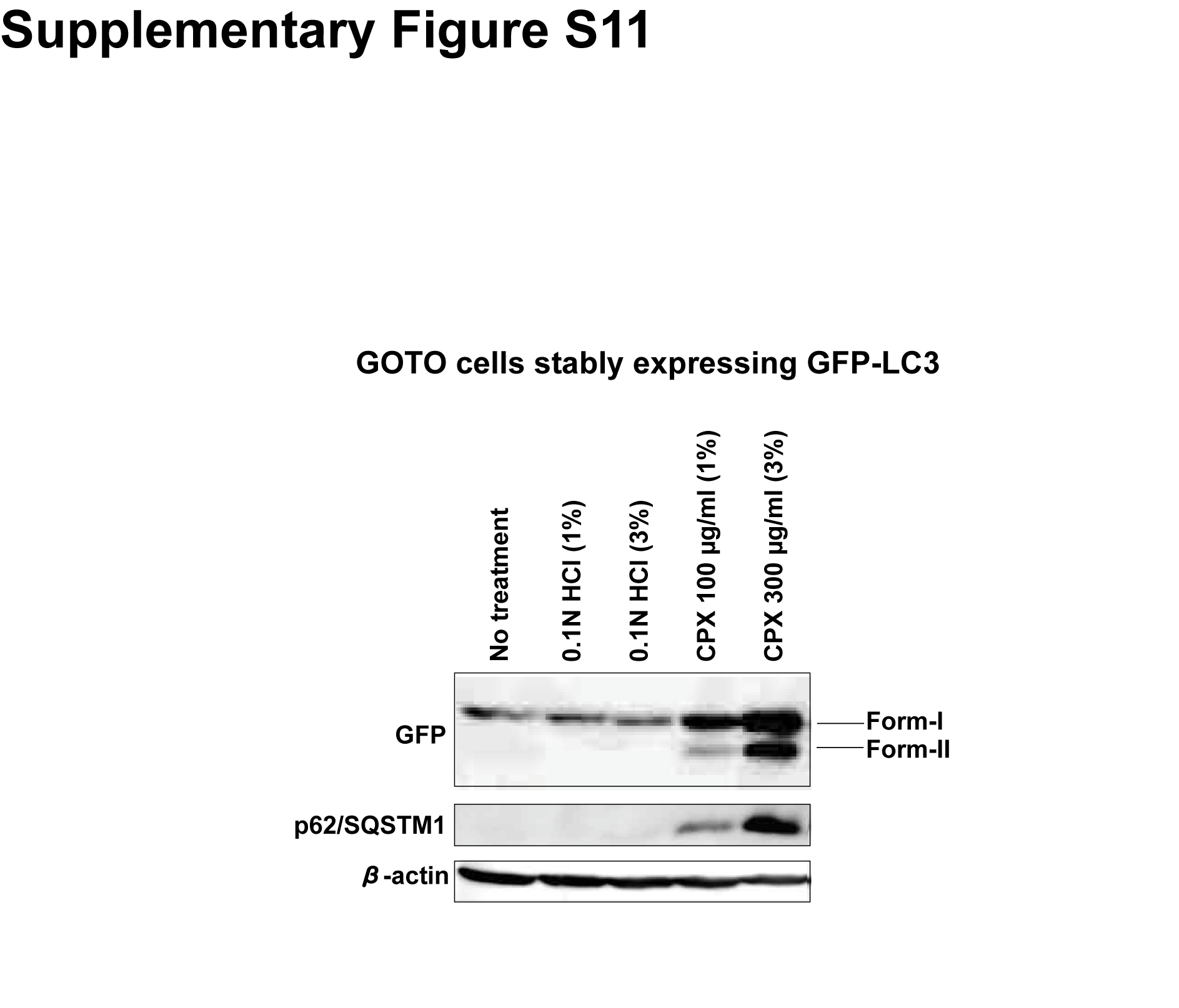

Supplement: Figure S11 — Detection of GFP-LC3 form-II and accumulation of p62/SQSTM1 on treatment with Ciprofloxacin (CPX) GOTO cells stably expressing GFP-LC3 (1×10^6^/well) were plated in 6-well plates, and treated with CPX (100 or 300 µg/ml) for one day. As a solvent for CPX, 0.1N HCl was added with 1 or 3% of the medium. Whole-cell lysate from treated cells was analyzed by immunoblotting with antibodies to the indicated proteins. (0.43 MB TIF) [file pone.0007099.s015.tif]

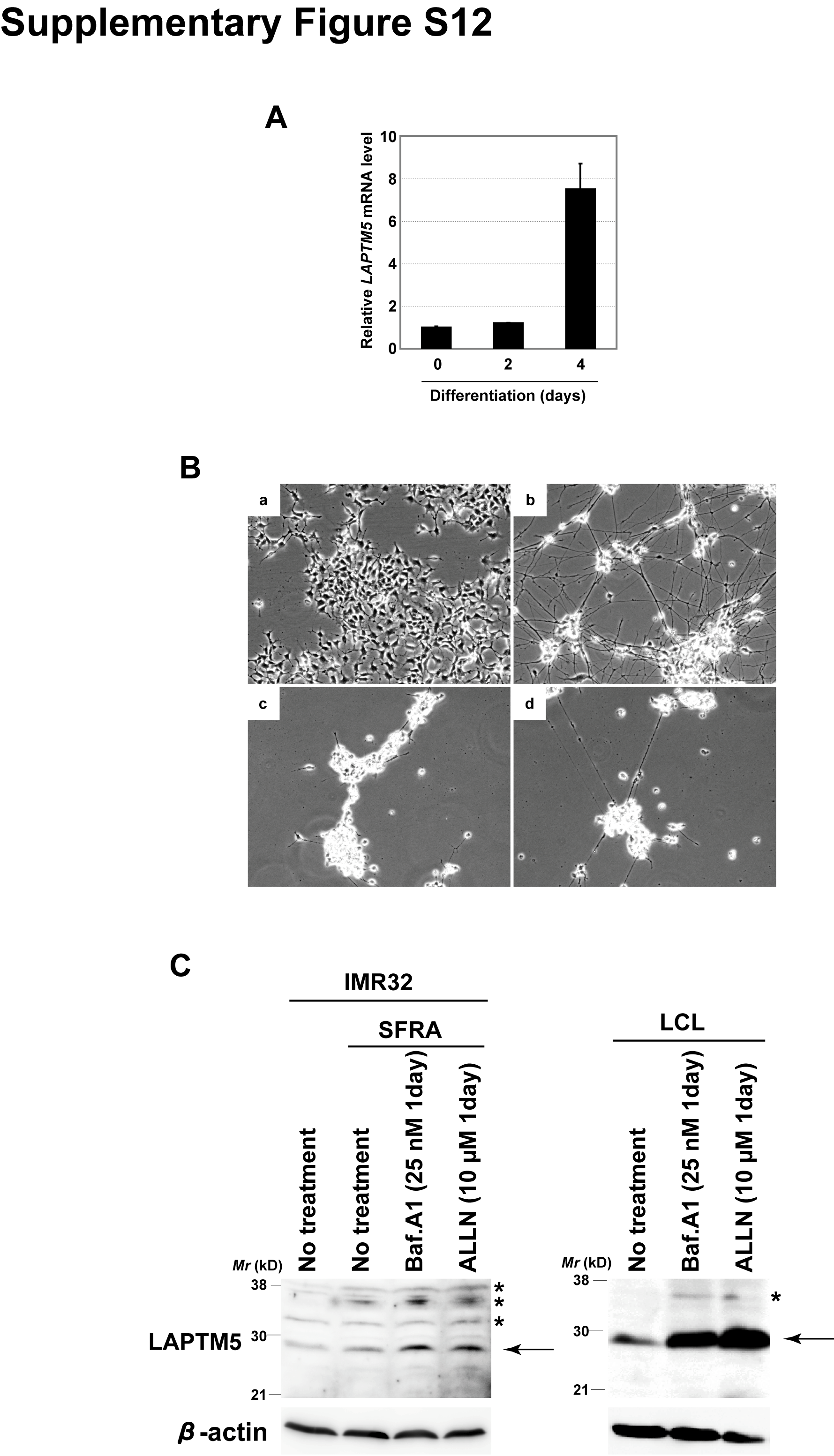

Supplement: Figure S12 — Detection of endogenous LAPTM5 by western blotting. (A) Relative levels of LAPTM5 mRNA levels during induction of differentiation in IMR32 cells. Cells were induced to neuronal differentiation by maintaining in serum-free medium including retinoic acid (RA,10 µM) for 4 days. The levels of LAPTM5 mRNA at the indicated days were determined by quantitative RT-PCR and normalized with each level of GAPDH mRNA. Vertical lines, SD. (B) Morphological change during RA-induced differentiation and treatment with lysosomal or proteasomal inhibitor. IMR32 cells (2×10^6^/well) were plated in 10 cm dishes and maintained in serum-free medium including RA (10 µM) (SFRA). At 3 days after induction, Baf.A1 (25 nM) or ALLN (10 µM) were added in medium and then cultured for 1 day. Treatment with SFRA efficiently induced the appearance of cells with a number of axonal outgrowth in IMR32 cells (a; parental IMR32 cells and b; differentiated IMR32 cells). Additional treatment with Baf.A1 (c) or ALLN (d) frequently induced cell death with a loss of axon. (C) Detection of endogenous LAPTM5 protein by western blotting. Cells were treated as indicated in (b). As a positive control for detection, whole-cell lysate from LCL highly expressing with or without Baf.A1 (10 nM, 1 day) or ALLN (10 µM 1 day) was used. Arrows indicate bands for endogenous LAPTM5 detected in expected size. TBS buffer including 0.05% Tween-20 and 1% Casein was used for blocking and reaction with antibody. Endogenous LAPTM5 was detected in expected size. Astarisks indicate non-specific bands. The results shown represent two independent experiments. (2.52 MB TIF) [file pone.0007099.s016.tif]
