# Supplementary material for: Lysosomal-Associated Protein Multispanning Transmembrane 5 Gene (LAPTM5) Is Associated with Spontaneous Regression of Neuroblastomas
Source: PLoS One. 2009 Sep 29;4(9):e7099. doi: 10.1371/journal.pone.0007099 (PMC2746316; doi:10.1371/journal.pone.0007099)
Supplement: Supplementary file 3 [file pone.0007099.s002.doc]

Supplementary Table 1 List of BACs containing possibly methylated regions in NB cell lines compared to PBMNCs by BAMCA analysis

BACs	Chr. band (distance;cM)[a]	STS	BAMCA ratio[b] GOTO 1	GOTO 2	IMR32 1	IMR32 2	Genbank No.	Symbol	Down-regulation of expression in both cell lines[c]	Differentially methylated *Sma* I sites[d]	Methylation status in NB tumors[e]
**PBMNCs vs. NB cell line**											
418B22	1p35.1 (30)	D1S1348	1.58	1.57	1.22	1.10	NM_002379	*MANT1*	no	no	-
							**NM_006762**	***LAPTM5***	**yes**	**yes**	**Methylated**

a; Distance from the top of short arm on chromosome 1.
b; BAMCA ratios were indicated by resulting for duplicate spots (1 and 2).
c; The presence ("yes") or absence ("no") of expression for each gene was determined by RT-PCR as indicated in Supplementary Figure S1.
d; The presence ("yes") or absence ("no") of differential methylation in SmaI sites for each gene was determined by MS-PCR as indicated in supplementary Figure S1.
e; The methylation status of LAPTM5 in primary NB tumors was determined by COBRA as indicated in supplementary Figure S1. "-" indicates "not tested".
