# Supplementary material for: Lysosomal-Associated Protein Multispanning Transmembrane 5 Gene (LAPTM5) Is Associated with Spontaneous Regression of Neuroblastomas
Source: PLoS One. 2009 Sep 29;4(9):e7099. doi: 10.1371/journal.pone.0007099 (PMC2746316; doi:10.1371/journal.pone.0007099)
Supplement: Supplementary file 4 [file pone.0007099.s003.doc]

Supplementary Table 2 Summary of copy-number aberrations for *MYCN*, *LAPTM5*, and 1p35 region in 13 NB cell lines

		Copy-number aberrations (by array-CGH & CGH analysis)[a]		Copy-number (by FISH analysis)[b]	
No.	cell lines	*MYCN* amplification	1p35 loss	**418B22 (*LAPTM5*)**	pUC1.77
1	GOTO	yes	yes	**1**	3
2	IMR32	yes	yes	**3**	5
3	SJ-N-CG	yes	no	**1**	6
4	CHP134	yes	no	**3**	2
5	MP-N-MS	yes	yes	**1**	2
6	KP-N-RT	yes	yes	**1**	2
7	SK-N-AS	no	no	**2**	3
8	SH-SY5Y	no	no	**2**	2
9	SK-N-SH	no	no	**2**	3
10	SJ-N-KP	no	no	**2**	3

a; Copy-number status for *MYCN* gene locus and 1p35 region was determined by array-CGH and conventional CGH analysis. "yes" indicates the presence of MYCN amplification or 1p35 loss, and "no" indicates the absence of them.
b; Two probes were used for FISH analysis. 418B22 (BAC clone), spotted on 1p36-contig array, is mapped on 1p35 and contain LAPTM5 gene locus. The pUC1.77 (plasmid) as a control is mapped on the pericentromeric region of chromosome 1.
