# Supplementary material for: Lysosomal-Associated Protein Multispanning Transmembrane 5 Gene (LAPTM5) Is Associated with Spontaneous Regression of Neuroblastomas
Source: PLoS One. 2009 Sep 29;4(9):e7099. doi: 10.1371/journal.pone.0007099 (PMC2746316; doi:10.1371/journal.pone.0007099)
Supplement: Supplementary file 5 [file pone.0007099.s004.doc]

Supplementary Table 3 Primer sequences

Applications	Forward (5'-3')	Reverse (5'-3')
Methylation analysys		
**Bisulfite sequencing & COBRA**		
LAPTM5-region-I	TGGTAGTGTTTTTTTAGTAAGT	AAAAATTCTTTCATTTATAAAAAA
LAPTM5-region-II	GTTAAATGGTTGGGGGTATTTTG	AACTTCACAAATTACCCAACAA
**Ms-SNuPE**		
CG-I site	TTTTTATTTTTTTTTTAGTTGTTT	
CG-II site	TGTTTC/TGTTTTAGTTGTGA	
**MS-PCR**		
SmaI-1	TTAACCACAGGCCCTGAGTC	GCTTCCAGCTCAGTCAAACC
SmaI-2	CAGAGAGGTGGAGAGCAGGA	CCCCTTCAGTGTCTCAGAGG
No. 1	TAGCAAAGGGTCCTGAAGTT	GCTCACTGCAAGCTCCGCCT
No. 2	AGACAGACGGGGGCCCCCTT	CCACACATCATATGCAGGTG
No. 3	CCTCCAGTGAGATGTGCATT	TGCCTGGGGTGTCCTTTCAC
No. 4	TGGCTTCTCTCCTCCATCCT	GACCCCTGGCTGCCTCTTTC
No. 5	GCCACCAGGGGCTCACTTGG	AGACGTCTGGCTGGCCAGGC
No. 6	ACACACCAAAGCCTGCCTGG	GCTGCAGTGAGCGATGAGCC
No. 7	CCAGACACACCCCCTCCCACC	TCCCTTAAGCCCCTCACCTC
No. 8	CAGGGCCAGGACTCAGGATT	CTGTGTCCAGTGGGGCTAGG
No. 9	ACAATGCCCACCTTCTGGGC	CGCCAGGAAAACGGCGTGAA
No. 10	GCTCACTGCAAGCTCCGCTT	GCAGGACGTGTCTGCGCGGG
No. 11	GGTCGCACAGCCAGGCGGCA	ACCCAGCCCCGGCCGGGCCT
No. 12	AACCTTAACAGCCCCACAGT	CAGATGGGGCCTGCGGTGAC
No. 13	GGGTGGGCCAAGCAAGGGCT	GTCTTGGGCAAGTCACAGTG
No. 14	GCAGAGGGGCAGCCTTGGCC	CCATGTGGTGAGTGTGCGGG
No. 15	CAAGAGGCCCAGGACACCAT	TGGTGTTGCAGCCCGGAACC
No. 16	GCTCACTGCAAGCTCCACCT	GGGCTTGAGATCCCGAGAGG
Expression analysis		
**TaqMan Probe for quantitative RT-PCR**		
TaqMan Probe	6-CCGTGCTAGCTCCTCCAAGTTCC-TAMRA	
**RT-PCR**		
LAPTM5	GTCCCTGCAAATCATGGACT	AGGTGGGCACTTCCATGTAG
MATN1	ATCGAGAAGCTGTCCAGGAA	GGATCCGTCAATGAGGAAGA
Construction		
**Expression vector**		
pAxCAwtit-LAPTM5	ATGGACCCCCGCTTGTCCAC	TCACACCTCTGAGTATGGG
